# A humanized mouse model of chronic COVID-19 to evaluate disease mechanisms and treatment options

**DOI:** 10.21203/rs.3.rs-279341/v1

**Published:** 2021-03-17

**Authors:** Esen Sefik, Ben Israelow, Jun Zhao, Rihao Qu, Eric Song, Haris Mirza, Eleanna Kaffe, Stephanie Halene, Eric Meffre, Yuval Kluger, Michel Nussenzweig, Craig B. Wilen, Akiko Iwasaki, Richard A. Flavell

**Affiliations:** 1 Department of Immunobiology, Yale University School of Medicine, New Haven, CT; 2 Section of Hematology, Yale Cancer Center and Department of Internal Medicine, Yale University School of Medicine, New Haven, CT; 3 Department of Pathology, Yale University School of Medicine, New Haven, CT, USA; 4 Laboratory of Molecular Immunology, The Rockefeller University, New York, NY, USA; 5 Department of Laboratory Medicine, Yale University School of Medicine, New Haven, CT,USA; 6 Howard Hughes Medical Institute, Yale University School of Medicine, New Haven, CT, USA

## Abstract

Coronavirus-associated acute respiratory disease, called coronavirus disease 2019 (COVID-19) is an infectious disease caused by severe acute respiratory syndrome coronavirus 2 (SARS-CoV-2). More than 90 million people have been infected with SARS-CoV-2 and more than 2 million people have died of complications due to COVID-19 worldwide. COVID-19, in its severe form, presents with an uncontrolled, hyperactive immune response and severe immunological injury or organ damage that accounts for morbidity and mortality. Even in the absence of complications, COVID-19 can last for several months with lingering effects of an overactive immune system. Dysregulated myeloid and lymphocyte compartments have been implicated in lung immunopathology. Currently, there are limited clinically-tested treatments of COVID-19 with disparities in the apparent efficacy in patients. Accurate model systems are essential to rapidly evaluate promising discoveries but most currently available in mice, ferrets and hamsters do not recapitulate sustained immunopathology described in COVID19 patients. Here, we present a comprehensively humanized mouse COVID-19 model that faithfully recapitulates the innate and adaptive human immune responses during infection with SARS-CoV-2 by adapting recombinant adeno-associated virus (AAV)-driven gene therapy to deliver human ACE2 to the lungs of MISTRG6 mice. Our unique model allows for the first time the study of chronic disease due to infection with SARS-CoV-2 in the context of patient-derived antibodies to characterize in real time the potential culprits of the observed human driving immunopathology; most importantly this model provides a live view into the aberrant macrophage response that is thought to be the effector of disease morbidity and ARDS in patients. Application of therapeutics such as patient-derived antibodies and steroids to our model allowed separation of the two aspects of the immune response, infectious viral clearance and immunopathology. Inflammatory cells seeded early in infection drove immune-pathology later, but this very same early anti-viral response was also crucial to contain infection.

## Introduction:

Coronavirus-associated acute respiratory disease, called coronavirus disease 2019 (COVID-19) is an infectious disease caused by a spillover of an animal coronavirus, identified and named as severe acute respiratory syndrome coronavirus 2 (SARS-CoV-2)^1^. The COVID19 pandemic has caused infection of over 90 million people and has claimed more than 2 million lives worldwide.

Prior to symptomatic recovery, an increased number of antibody-secreting cells and activated T cells are detected in patients, indicating substantial anti-viral immunity in non-severe COVID-19^2^. Yet, in severe COVID-19 robust inflammatory cytokine production coupled with decreased circulating lymphocytes and failure to generate germinal centers characterizes the immunopathology that leads to ARDS and multi-organ dysfunction^2–4^. Dysregulated myeloid and lymphocyte compartments, including monocytes, macrophages, neutrophils, NK cells and antibody secreting B cells, have been described as players in the observed lung immunopathology.

COVID19 is a remarkably heterogenous disease with few therapeutic options, amplifying the urgency to better understand mechanisms of immunopathology and immune-protection in this devastating disease. Disparities in the apparent efficacy of appropriate treatments of COVID-19 in patients renders accurate model systems essential to rapidly and comprehensively evaluate promising discoveries. Here we present a comprehensively humanized COVID-19 model and evaluate its potential to faithfully model the innate and adaptive human immune system during infection with SARS-CoV-2.

Mice with a human immune system (humanized mice) serve as invaluable tools to study the development and function of the human immune system in vivo. Humanized mice are generated by transplantation of human hematopoietic stem and progenitor cells (HSPCs) into various strains of immune-compromised mice that thereby allow xeno-graftment^5,6^. The MISTRG6 mouse model was engineered by a human/mouse homolog gene-replacement strategy to provide physiological factors with regard to quantity, location and time and enable essentially all classes of human hematopoietic cells to develop in mice. MISTRG6 (acronym for genes replaced) mice encode humanized **M**-CSF (enabling monocytes and tissue macrophage development)^7^, GM-CSF/**IL**-3 (to provide lung alveolar macrophages)^8^, **S**IRPα (establish macrophage tolerance to human cells )^9^, **T**hPO (hematopoiesis and platelets)^10^, and IL**6** (better engraftment allowing study of adult human patients and improved antigen-specific antibody responses as well as human IL-6 per se^11,25,12,13,^ in a **R**ag2/**G**amma common chain deleted background. MISTRG6 mice have a comprehensive immune system relatively comparable to humans in strong contrast to other commonly used humanized mouse models, which either lack relevant human myeloid cells, specifically monocytes and alveolar macrophages, or support human myelopoiesis but at the expense of human hematopoietic stem cell maintenance ^14,15^. Of particular importance for COVID-19 research, MISTRG mice, express physiologic levels of GM-CSF, essential to repopulation of the murine lung with human alveolar macrophages ^16^; and M-CSF which enable tissue macrophage and blood monocyte development^7^, which together in humans are thought to contribute to COVID-19 severity. Moreover, the human myeloid cells secrete IL-15 which directs the robust development of human NK cells that are also implicated in COVID19 pathogenesis^17^. By adapting recombinant adeno-associated virus (AAV)-driven gene therapy to deliver human ACE2 to the lungs^18^, which allows infection with SARS-CoV-2 of MISTRG6 mice engrafted with a human hematopoietic stem and progenitor cells, we created a humanized mouse model of COVID19 that recapitulates the distribution and function of the human innate and adaptive immune system and is amenable to the mechanistic study of COVID 19 and its myriad of complications ^12,19^.

## Results:

### MISTRG6 humanized mice that transiently express hACE2 can be infected with SARS-CoV-2.

SARS-CoV-2 does not infect standard laboratory mice due to differences between mouse and human ACE2 receptor that limit viral entry^20,21,^. Introduction of human ACE2 into the murine host lung via recombinant adeno-associated virus (AAV)-driven gene therapy enables SARS-CoV-2 infection; however, in a standard laboratory mouse AAV-mediated hACE2 expression affords only acute, transient infection with SARS-CoV-2. We hypothesized that a functional human immune system would confer much of the chronicity and pathology seen in patients onto a small animal model^22,23^.

**Figure 1.**
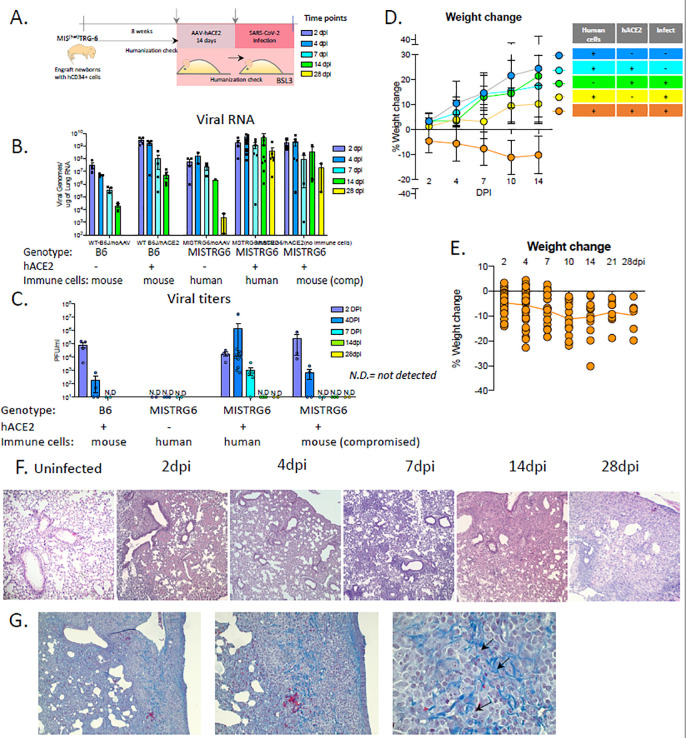
MISTRG6 humanized mice that transiently express hACE2 can be infected with SARS-CoV-2. **A.** Schematic of experimental design. **B.** Viral RNA (quantification of N gene) **C.** Viral titers measured by PFU in homogenized lung tissue at 2, 4, 7 14, 28 days post infection (dpi) in B6 control or reconstituted or unengrafted MISTRG6 mice expressing or lacking human ACE2. N=2–24. Means with SD of at least 2 independent experiments are presented. **D.** Weight change during the course of infection plotted as percent change compared with original weight measured just before inoculation with SARS-Cov2. N=6–37. Means with SD of at least three independent experiments are presented. **E.** Weight change of individual mice reconstituted MISTRG6-hACE2 mice on 2,4,7,10,14,21 and 28dpi. N=6–37. **F.** Histopathology by H&E staining of infected (2, 4, 7, 14 and 28dpi) or uninfected lungs. N=4–8 per time point. Representatives of at least three independent experiments are presented. **G.** Trichrome staining of infected lungs at 28dpi. Arrows indicate areas with Collagen deposition. N=4–8 per time point. Human ACE2 was delivered by AAV to lungs of wild type or reconstituted adult MISTRG6 mice. Mice were then infected (or left uninfected) intranasally with SARS-CoV-2 and weighed on 0, 2, 4, 7, 14, 21 and 28 dpi.

We successfully delivered AAV-hACE2 to lung tissues^18^ of immune-reconstituted MISTRG6 mice (MISTRG6-hACE2) (Fig 1A). MISTRG6-hACE2 mice were then infected with SARS-CoV-2 which yielded comparable viral RNA levels and viral titers (Fig. 1B–C) 2 as described for wild type mice early in infection but with higher viral titers which were sustained for at least 7 days as well as chronically high levels of viral RNA for at least 28 days post infection (dpi). In contrast, MISTRG6 mice lacking AAV-hACE2 expression did not have detectable viral titers even early in infection, confirming the necessity of human ACE2 for infection (Fig. 1C). Laboratory mice do not normally develop severe, chronic disease^18,24,25^. In contrast, the presence of human immune cells in MISTRG6-hACE2 mice caused more severe disease with severe weight loss and a chronic inability to restore body weight for at least 28 dpi (Fig. 1D). MISTRG6 humanized mice also exhibit more severe lung pathology compared to published wild type mice, and other animal models^18,25–28^. This lung pathology was characterized by infiltrating monocytes and macrophages and unresolved diffuse alveolar damage, reminiscent of human patients^4,29–31^ (Fig. 1F–S1A). Strikingly, severe lung pathology persisted for at least 28dpi (Fig S1A), by which time signs of fibrosis manifested (Fig. 1G). Of note, MISTRG6-hACE2 mice that were infected but not engrafted with human HSPCs (so lack human immune cells), had reduced viral titers (Fig. 1B–C). Thus, these data suggest the human immune cells contribute to worsened pathology and amplification of viral infection.

### Immune landscape in MISTRG6-hACE2 humanized mice infected with SARS-CoV2 is characterized by inflammatory macrophages and monocytes.

**Figure 2.**
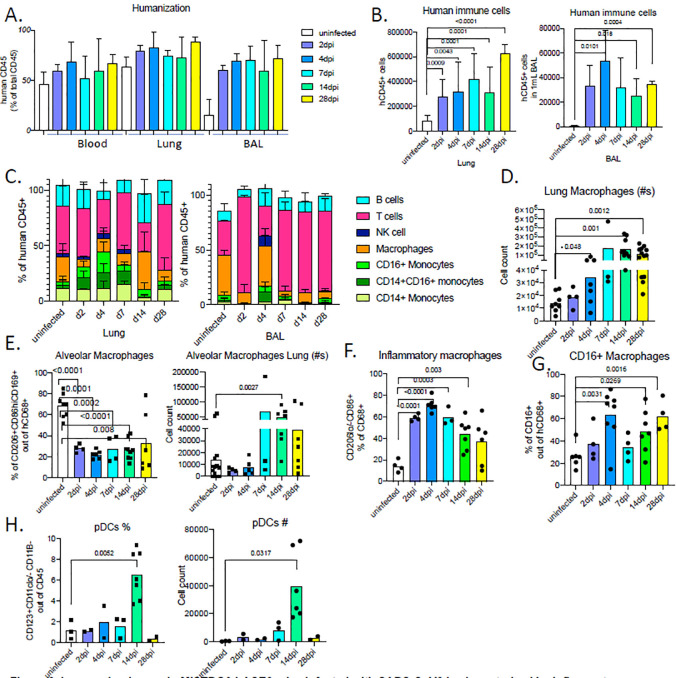
Immune landscape in MISTRG6-hACE2 mice infected with SARS-CoV2 is characterized by inflammatory macrophages and monocytes. **A.** Humanization measured by ratio of human CD45+ cells in total CD45+ cells (mouse and human CD45+ combined) in blood, lung and BAL of uninfected and infected MISTRG6-hACE2 mice. N=4–12. **B.** Human immune cell numbers in lungs and BAL of uninfected and infected mice at 2,4,7,14,28 dpi. N=4–12. Unpaired, two-tailed t-test. Only P-values <0.05 are plotted. **C.** Human immune lineages lungs and BAL of uninfected and infected mice at 2,4,7,14,28 dpi within the human CD45+ population. Classical monocytes (CD14+), Intermediate monocytes (CD14+CD16), non-classical monocytes (CD16+CD14−), macrophages (CD68+), NK cells (NKP46+), T cells (CD3+), B cells (CD19+ and/or CD20+). N=4–12. Means with SD are plotted. **D.** Number of human macrophages in lungs of uninfected and infected mice at 2,4,7,14,28 dpi. N=4–10. Unpaired, two-tailed t-test. **E.** Frequency and number of human alveolar macrophages marked by CD206hi, CD86+, CD169+ expression within the hCD45+CD68+ population in the lungs of uninfected and infected mice at 2,4,7,14,28 dpi. N=3–10. Unpaired, two-tailed t-test. **F.** Frequency of inflammatory human lung macrophages marked by CD206-/lo, CD86hi macrophages within the hCD45+CD68+ population in the lungs of uninfected and infected mice at 2,4,7,14,28 dpi. N=3–10. Unpaired, two-tailed t-test. P values<0.05 plotted. **G.** Frequency of CD16+ human lung macrophages marked by CD16+ macrophages within the hCD45+CD68+ population in the lungs of uninfected and infected mice at 2,4,7,14,28 dpi. N=4–8. Unpaired, two-tailed t-test. P values<0.05 plotted **H.** Frequency and number of human pDCs marked by CD123+ CD11b− CD11c−/lo cells withing hCD45+ population in the lungs of uninfected and infected mice at 2,4,7,14,28 dpi Means of at least 3 independent experiments are presented. Mean with SD or individual values are plotted.

We next characterized human immune cells in SARS-CoV2 infected and uninfected control MISTRG6-hACE2 humanized mice by flow cytometry to better evaluate the immunological landscape that drives immunopathology and viral clearance (Fig S2). The degree of humanization, measured by the ratio of human immune cells among total immune cells, was comparable in blood and lung between uninfected and infected mice, but SARS-CoV2 infection drastically increased recruitment of human immune cells to the lower respiratory tract and lung parenchyma, as assessed by the number of human cells in whole lung homogenates and bronchiolar lavage (BAL) (Fig. 2A–B). The infiltrates present in lungs and bronchioalveolar lavage at day 2–4 post infection consisted of human monocytes, macrophages and T cells (Fig. 2C), again reminiscent of human COVID-19 lung pathology.

The lung monocytes of infected mice comprised all three monocyte subsets (CD14+ classical; CD14+CD16+ intermediate; and CD16+ non-classical; Fig. 2A–C). In line with published characterization of monocytes from healthy human lungs^32^; uninfected humanized mouse lungs harbored only classical CD14+ monocytes which expanded in response to SARS-CoV2 infection. Intermediate and non-classical monocytes infiltrated infected lungs at high frequencies as early as 2dpi, peaking at 4dpi. Increased numbers of macrophages were observed at 4dpi, which remained high until 28dpi, the last time point measured (Fig. 2D). By 2dpi, the macrophage compartment was already enriched for inflammatory and monocyte-derived macrophages, which outnumbered alveolar macrophages, suggesting that macrophages seeded early from the circulation are the long term contributors of immunopathology (Fig. 2E–G). To our surprise, plasmacytoid dendritic cells (pDCs), known for their contribution to early antiviral response and main producers of type I interferon alpha (IFNα), were particularly enriched but substantially later in infection (14dpi in Fig. 2H). This coincided with a chronically sustained interferon response coupled with sustained inflammatory macrophages, which is in line with observations in patients with severe COVID19 immunopathology that is believed to be at least partially driven by inflammatory macrophages and accompanied by a sustained type I interferon response ^33,34^.

### COVID-19 in MISTRG6-hACE2 humanized mice presents with systemic T cell lymphopenia.

**Figure 3.**
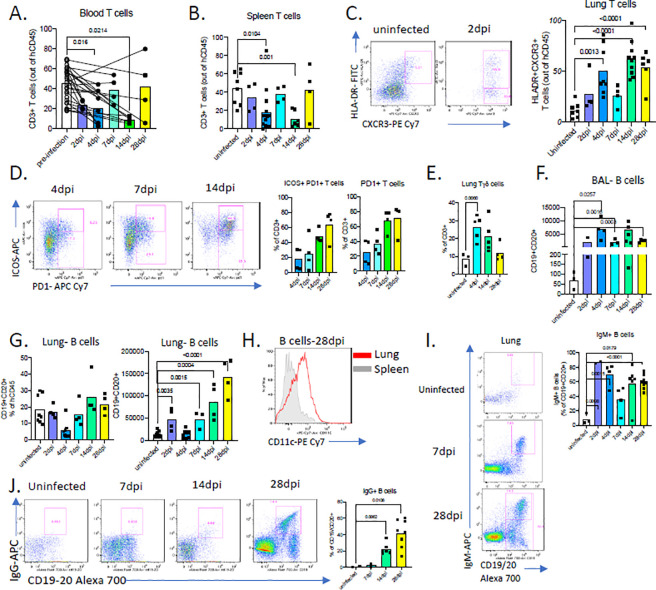
COVID-19 in MISTRG6 presents with systemic T cell lymphopenia. **A.** Frequencies of human CD3+ T cells within human CD45+ population in the blood pre and post-infection (2,4,7,14,28dpi). Lines connect pre and post-infection values for the same mouse. Paired, two-tailed t-test. N=4–6 per infection time point. P-values<0.05 are plotted. **B.** Frequencies of human CD3+ T cells within human CD45+ population in the spleens of uninfected and infected mice at 2,4,7,14,28 dpi. Mean of at least three independent experiment. Unpaired, two-tailed t-test. N=4–14 per infection time point. P-values<0.05 are plotted. **C.** Representative flow cytometry plots of HLA-DR and CXCR3 expression on human lung T cells and frequencies of HLA-DR+CXCR3+ lung T cells in uninfected and infected mice at 2,4,7,14,28dpi. N=4–10. Unpaired, two-tailed t-test. P-values<0.05 are plotted. **D.** Representative flow cytometry plots of ICOS and PD1 expression on human lung T cells and frequencies Icos+ PD1+ or PD1+ T cells in uninfected and infected mice (4,7,14,28dpi). N=4. **E.** Frequencies of TCRgamma/delta T cells among human lung T cells in uninfected and infected mice (4,14,28dpi). N=3–5. P-values<0.05 are plotted. **F.** Numbers of human B cells in the BAL of uninfected or infected mice at 2,4,7,14,28 dpi. N=3–6. Unpaired, two-tailed t-test. P-values<0.05 are plotted. **G.** Frequencies and numbers of human B cells within hCD45+ population in the lungs of uninfected or infected mice at 2,4,7,14,28 dpi. N=4–8. Unpaired, two-tailed t-test. P-values<0.05 are plotted. **H.** CD11 c expression on CD19+ B cells from spleen and lungs of infected mice at 28dpi. N=4 **I.** Representative flow cytometry plots and frequencies of IgM+ B cells in the lungs of uninfected and infected mice 2,4,7,14,28dpi. N=2–8 **J.** Representative flow cytometry plots and frequencies of IgG+ B cells in the lungs of uninfected and infected mice 7,14,28dpi. N=2–8

**Figure 4.**
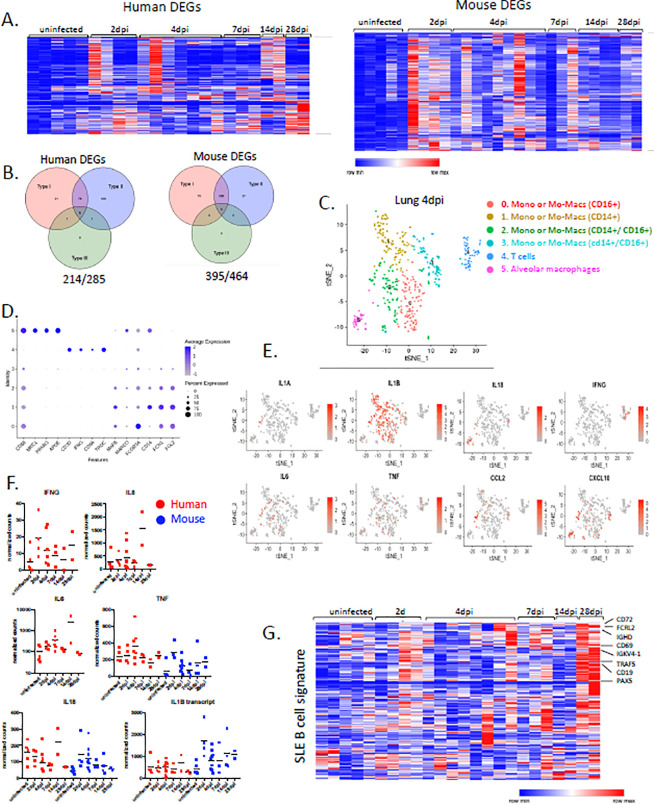
SARS-CoV-2 lungs sustain interferon responsive gene signatures and display an SLE like features **A.** Heatmap of differentially regulated human and mouse genes (combined list of genes Log2, FoldChange >1 in each infected time point vs uninfected lungs; adjusted p value<0.05; mean normalized count>5). Transformed normalized counts in lungs of uninfected or infected MISTRG6-hACE2 plotted over the course of infection were clustered using Spearman Correlation. Row min and max of transformed values, calculated by subtracting row mean and diving by STD for each gene across all samples, are visualized. **B.** Distribution of interferon responsive genes within human and mouse DEGs **C.** t-distributed stochastic neighbor embedding (*t*-SNE) plot with clustering results of single cell RNA sequencing of human immune cells from lungs at 4dpi. Single cell suspensions from whole infected lung at 4dpi were processed and sequenced. There were 421 cells identified as human immune cells. **D.** Expression of cluster identifying genes in human immune cells described in C. **E.** Cluster distribution and expression of human inflammatory cytokines for clusters described in C-D. **F.** Normalized counts for inflammatory cytokines implicated in COVID19 patients. Counts were reported separately for human(red) and mouse(blue) cytokine genes. **G.** Heatmap of genes that are implicated in SLE like B cells based on GSE10325 (Hutcheson et al., 2007) in infected lungs of MISTRG6 mice at 2,4,7.14.28 dpi. Row min and max of transformed values, calculated by subtracting row mean and diving by STD for each gene across all samples, are visualized.

A notable characteristic of human COVID-19 is profound T cell lymphopenia and this is strikingly recapitulated in our humanized mice. Infected MISTRG6-*hACE2* humanized mice also presented with lymphopenia characterized by a profound loss of T cells, especially CD8+ T cells, in blood (Fig. 3A; Fig. S3A–B) and spleens (Fig. 3B, Fig. S3B–C) as observed in COVID-19 patients^35^ Moreover, in the adaptive arm of the anti-viral response in the lung of *MISTRG6-hACE2 humanized mice,* T cells, the main producers of IFN- gamma, displayed markers of activation such as HLA-DR, CXCR3 (Fig. 3C), ICOS and PD1 (Fig. 3D) as reported in COVID-19 patients^35–37^. T cell populations comprised of both TCRalpha/beta T cells (Tαβ) that were enriched for CD4+ T cells, and TCRgamma/delta T cells (Tγδ) cells in both uninfected and infected mice (Fig. S3D). Yet, during the course of infection, as the circulating T cell numbers decline, the total number of T cells in lungs increased with higher representation of Tγδ cells compared with uninfected mice (Fig. 3E). The early T cell response in infected lungs corresponded to an increase in both resident and infiltrating Tγδ cells (as assed by scRNA seq; cluster 4 in Fig 4C) as well as bystander activated memory Tαβ cells as determined by the lung transcriptional profile (Fig. S4A), B cell numbers also gradually increased in response to infection over several weeks, peaking late at 28dpi in lungs and BAL (Fig. 3G–H). Although the germinal center B cell response has been reported to be suboptimal in reconstituted MISTRG6 humanized mice^13^,a high proportion of IgM+ B cells were seen early in infection that were subsequently replaced by IgG+ B cells later in infection (14dpi and 28dpi) and thus defined the anti-viral B cell response (Fig. 3I–J, Fig. S3E). B cells, particularly late in infection (14dpi and 28dpi), expressed high levels of CD11c (Fig 3H, S3F), implicating a highly inflammatory, SLE-like extrafollicular B cell response^38,39^ in humanized COVID19, reminiscent of the human disease^40^. In contrast to T cell lymphopenia, B cell numbers in the humanized mice were normal or even increased (Fig 3F–G; Fig. 3SG) as is also seen in the majority of patients ^41^. A robust memory B-cell expansion is detected early in human infection with secretion of serum IgM and IgA antibodies detected earlier than IgG^30^. Humans infected with SARS-CoV-2 have initial serum IgM and IgA titers which decline (~28 days), as IgG titers peak (~49 days)^42^. We observe a similar dynamic for IgM+ and IgG+ B cells in humanized lungs with IgM+B cells declining over time and IgG+ B cells emerging as early as 14dpi but reaching high levels at 28dpi (Fig. 3 I–J; S3E,H). Thus, MISTRG6-hACE2 humanized mice recapitulate T and B cell responses induced by SARS-CoV-2 infection in COVID-19 patients.

### Transcriptional landscape of SARS-CoV-2 infection is marked by sustained interferon response and SLE like features, reminiscent of COVID-19 patients.

Next, we evaluated the transcriptional landscape in uninfected and SARS-CoV-2 infected lungs of humanized mice at multiple time points (2, 4, 7, 14, 28 dpi). Mapping of transcripts to the human genome or the mouse genome separately identified 285 human genes and 516 mouse genes that were over-represented in infected lungs (Fig. 4A, Table S1). Although there was marked heterogeneity in the strength of the response, the anti-viral response was strikingly sustained throughout the course of infection long after infectious virus was apparently cleared, suggesting that early anti-viral responses were either maintained or amplified late in infection (Fig. 4A). Corresponding pathway analysis of these differentially expressed mouse genes (DEGs) using multiple platforms (Gene Ontology (GO), Gene Set Enrichment analysis (GSEA), Ingenuity) identified cellular response to interferons, cytokine production, ribonuclease activity and neutrophil activation as top biological processes that are induced during SARS-CoV-2 infection in humanized mice (Table S2). A similar analysis showed human genes that were enriched for extracellular matrix assembly, opsonization, complement activation with a focus on immune phenotypes in monocytes, activated T cells and B cells, which further corroborated our findings from flow cytometric analysis (Table S2). Similarly, circuitry of monocytes, macrophages, activated T cells and in particular extrafollicular B cells has been described in humans with SARS-CoV-2 pneumonia^43^.

We performed single cell RNA sequencing using the 10X Genomics platform to better evaluate the phenotype of human cells in lungs of infected MISTRG6-hACE2 humanized mice. Our transcriptome analysis of human cells at the single cell level also revealed that human macrophages, monocytes and T cells were abundant in the lung at 4dpi (Fig. 4C–D). Monocytes that have infiltrated the infected lung tissue were already differentiating into macrophages at this stage of infection and these infiltrating monocytes and macrophages (clusters 0,1,2,3) were the main producers of inflammatory cytokines IL-1A, IL-1B, TNF and IL-6, which have been shown to be elevated in severe COVID-19 patients^36,37^ (Fig. 4E–F). In line with recent studies that characterize human alveolar macrophages and T cells in COVID-19^43^, alveolar macrophages in infected MISTRG6-hACE2 humanized mice were the main producers of T cell chemoattractants such as CXCL10 (Fig. 4E).

Strikingly, the majority of the human and mouse DEGs in lungs were interferon responsive genes (Fig. 4B). Although type I and type III interferon expression *perse* could not be detected, interferon responsive genes were sustained at high levels throughout infection persisting even as late as 14dpi and 28dpi, recapitulating the interferon-dependent phenotypes identified in COVID19 patients (Fig. 4A–B, S4A, Table S3) ^30,33,44,45^. IFN signaling coinciding with the onset of lung recovery (7–9 dpi) in influenza infection has been shown to prevent epithelial cell proliferation and differentiation, hence interfering with lung repair^46^. In line with known effects of interferons on lung tissue recovery during influenza infection^46^, our findings suggest that sustained type I IFN signaling may also contribute to sustained lung tissue injury in COVID19 as supported by histopathological assessment of 14 and 28 dpi lungs of the infected humanized mice (Fig. 1F). Human and mouse DEGs were also enriched for type II responsive genes. Type II interferon IFNg was mainly produced by T cells (Fig. 4C,E) starting as early as 2dpi and was sustained until 28dpi. (Fig. 4F). In addition, levels of various pro-inflammatory cytokines (IL6, IL8,TNF, IL1B) were elevated and peaked late in infection (IL6 and IL8 at 14dpi) after viral clearance, further supporting that delayed immune-resolution is a characteristic of humanized COVID-19 (Fig 4F). Inflammatory cytokine signature, particularly elevated IL6 and IL8 but not TNF, closely correlated with severity of COVID19 in patients^47,48^ and is notably recapitulated in humanized COVID-19.

We focused analysis on the genes that typify bystander activation of memory T cells^49^; this suggested that early in infection, T cell activation in 2dpi and 4dpi lungs may be an antigen-independent, interferon driven response (Fig. S4A). We sought to identify the origin of genes upregulated in patients by validating their expression in our infected mice and then identifying their cellular source. This showed that the B cell response in humanized lungs was particularly enriched for genes that are upregulated in moderate and severe COVID19 compared with healthy donors (Fig. S4B–C). Strikingly, genes differentially expressed at 28dpi (our latest timepoint) were enriched for extrafollicular differentiation of B cells and also presented with features of systemic lupus erythematosus (SLE) pathways ((Fig. 4G). Lack of germinal center formation in the spleens and lymph nodes of patients that have succumbed to COVID-19 coupled with extrafollicular B cell responses have been correlated with morbidity and poor clinical outcomes in COVID19 patients ^40,50^. In line with these observations, unbiased pathway analysis of the 28dpi lung transcriptome (Fig. S4B) and a more focused look at the SLE gene signatures ^51^ identified a particular enrichment of SLE-like extrafollicular responses in lungs of infected mice by 28dpi (Fig. 4D). Furthermore, B cell responses at 28dpi exhibited the features of bystander responses previously characterized in influenza infection in humans^52^ (Fig. S4D), further suggesting a highly inflammatory, bystander B cell response in humanized COVID19^36,37^. Taken together, our transcriptome analysis of infected humanized lungs identifies monocyte derived-macrophages at the center of early anti-SARS-Cov-2 response that maintain an interferon dependent response that is further amplified later in infection. Bystander T cell activation and SLE-like features of B cells suggest that interferons not only shape innate immunity but also impact the nature of the adaptive immune response induced by SARS-CoV-2.

### Human monoclonal recombinant antibodies as prophylactic and therapeutic interventions impact disease outcome.

**Figure 5.**
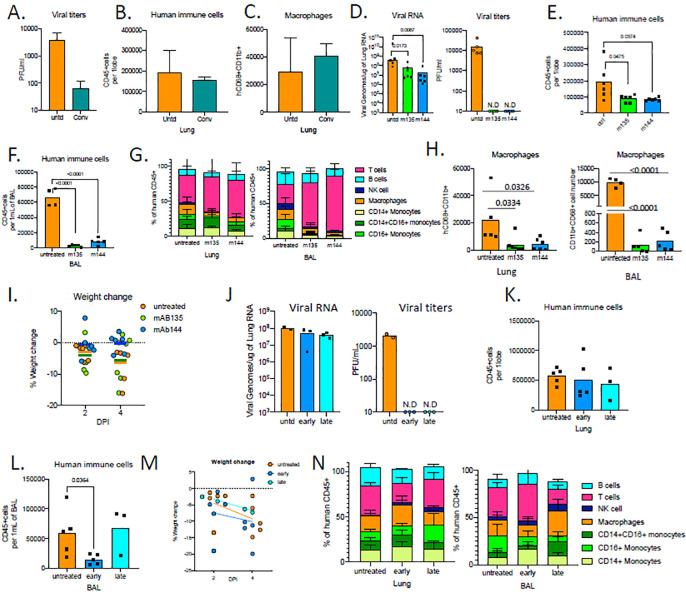
Human monoclonal recombinant antibodies as prophylactic and therapeutic interventions impact disease outcome. **A.** Viral titers measured by PFU in homogenized lung tissue at 4 dpi in MISTRG6-hACE2 mice that received prophylactic treatment of convalescent patient plasma or left untreated. N=2–4 **B.** Human immune cells at 4 dpi in lungs of MISTRG6-hACE2 mice that received prophylactic treatment of convalescent patient serum or left untreated. N=2–4 **C.** Human macrophages (hCD45+ hCD68+) at 4 dpi in lungs of MISTRG6-hACE2 mice that received prophylactic treatment of convalescent patient serum or left untreated. N=2–4 **D.** Viral RNA and viral titers measured by PFU in homogenized lung tissue at 4 dpi in MISTRG6-hACE2 mice that received prophylactic treatment of monoclonal antibody clone 135 (m135) or clone 144(m144) 8 hours prior to infection or left untreated (untd). N=4–6. Mann-Whitney, two-tailed test. **E.** Human immune cells in lungs of MISTRG6-hACE2 mice received a prophylactic treatment of monoclonal antibody clone 135 (m135) or clone 144(m144) 8 hours prior to infection or left untreated (untd). N=5–6 **F.** Human immune cells in BAL of MISTRG6-hACE2 mice received a prophylactic treatment of monoclonal antibody clone 135 (m135) or clone 144(m144) 8 hours prior to infection or left untreated (untd). N=4–6 **G.** Human immune lineages lungs and BAL of mAb treated or untreated mice at 4 dpi within the human CD45+ population. Classical monocytes (CD14+), Intermediate monocytes (CD14+CD16), non-classical monocytes (CD16+CD14-), macrophages (CD68+), NK cells (NKP46+), T cells (CD3+), B cells (CD19+ and/or CD20+). MISTRG6-hACE2 mice received a prophylactic treatment of monoclonal antibody clone 135 (m135) or clone 144(m144) 8 hours prior to infection or left untreated (untd). N=4–6 **H.** Human macrophages (hCD45+ hCD68+) at 4 dpi in lungs and BAL of MISTRG6-hACE2 mice that received prophylactic treatment of mAbs (clone 135 or 144) or left untreated. N=4–6 **I.** Weight change in mAb treated mice (prophylaxis) at 2days and 4days post-infection plotted as percent change compared with original weight measured just before inoculation with SARS-Cov2. N=4–6 **J.** Viral RNA and viral titers measured by PFU in homogenized lung tissue at 4 dpi in MISTRG6-hACE2 mice that received post infection treatment of a mixed cocktail of monoclonal antibodies clone 135 (m135) and clone 144(m144) or left untreated (untd). Early treatment groups were treated 11hours post-infection and late treatment 35 hours post-infection. **K.** Human immune cells in lungs of MISTRG6-hACE2 mice that received early, late or no treatment of monoclonal antibody mix. Unpaired, two-tailed t-test. N=3–5. P-values<0.05 are plotted. **L.** Human immune cells in BAL of MISTRG6-hACE2 mice that received early, late or no treatment of monoclonal antibody mix. Unpaired, two-tailed t-test. N=3–5. P-values<0.05 are plotted. **M.** Weight change upon mAb therapeutic treatment at 2days and 4days post-infection plotted as percent change compared with original weight measured just before inoculation with SARS-Cov2. N=3–5. **N.** Human immune lineages lungs and BAL of mAb treated or untreated mice at 4 dpi within the human CD45+ population. Classical monocytes (CD14+), Intermediate monocytes (CD14+CD16), non-classical monocytes (CD16+CD14−), macrophages (CD68+), NK cells (NKP46+), T cells (CD3+), B cells (CD19+ and/or CD20+). MISTRG6-hACE2 mice received a prophylactic treatment of monoclonal antibody clone 135 (m135) or clone 144(m144) 8 hours prior to infection or left untreated (untd). MISTRG6 mice were engrafted neonatally with CD34+ cells isolated from at least 2 donors. Pooled, infection matched representative results of at least 2 independent experiments are presented. P-values<0.05 are plotted. Mean with SD or individual values are plotted.

We wanted to test whether ***MISTRG6-hACE2*** mice could be used to evaluate patient-derived human antibodies as modulators of infection. Convalescent plasma samples from the top 30 neutralizers in a cohort of 148 individulas were pooled to create a mixture with an NT50 titer of 1597 against HIV-1 pseudotyped with SARS-CoV-2 S protein^23^. ***MISTRG6-hACE2*** mice were treated with the mixed plasma 8 hours before infection with SARS-CoV-2(Fig. 5A). The treated mice had significantly lower viral titers in lungs at 4dpi and therefore the plasma was only partially effective (Fig 5A). Yet, prophylactic convalescent plasma did not prevent human immune cell infiltration, particularly inflammatory macrophages, to the lungs (Fig. 5B–C) or weight loss(Fig. S5B). These findings highlight the partial efficacy of prophylactic administration of convalescent plasma^22,53,54^. Sequencing the antibody genes from infected humans has revealed the expansion of closely related Receptor Binding Domain of the Spike protein (RBD)-specific B cell antibody clones in different SARS-CoV-2 infected individuals. Although most convalescent plasma samples obtained from patients who recovered from COVID-19 did not contain high levels of neutralizing activity, RBD-specific antibodies with potent antiviral activity were found in all individuals tested^23^.^23^. Monoclonal recombinant antibodies (mAbs) cloned from these patients had high neutralizing activity against SARS-CoV-2 *in vitro* and *in vivo* in mouse adapted SARS-CoV-2 infection ^23,55^. Thus, we tested two complementary mAb clones in vivo for prophylactic and therapeutic treatments of SARS-CoV-2 infection in humanized mice (Fig. S5A,F). Mice were either treated with individual mAb prior to infection, or with the two mAbs combined, at two time points post infection (11 hours and 35 hours post infection) and analyzed at 4dpi for characterization of disease parameters and immune cells (Fig. S5A,F). As measured by viral titers and viral RNA in lungs, prophylactic treatment with mAbs prevented SARS-CoV-2 infection (Fig. 5D) Prophylactic antibody administration also attenuated immune infiltration, characterized by fewer infiltrating immune cells, in particular decreased macrophages in lungs and BAL of mAb-treated mice (Fig. 5E–H, S5C). Strikingly, treatment with mAb clone 144 also prevented systemic T cell lymphopenia (Fig. S5D–E) and weight loss (Fig. 5I). Next, we tested whether MISTRG6 mice could be used to model therapeutic mAb therapy. While therapeutic treatment of mAbs similarly prompted infectious viral clearance at both early (11hpi) and late time points (36hpi; Fig. 5J), by contrast to prophylaxis, mAbs failed to prevent immune infiltration in lungs (Fig. 5K). Humanized mice treated with both mAbs early (11h) post infection had fewer immune cells in BAL at 4dpi compared to untreated and late (35h)-treated mAb groups, suggesting that the immune-infiltrate and inflammatory responses are attenuated when mice are treated with mAbs early but less so when treated later in infection (Fig. 5L). Although neither therapeutic intervention prevented weight loss, early treatment prevented systemic T cell lymphopenia (Fig. 5M, S5G). By contrast, later administration of neutralizing mAbs showed little effect, and a similar infiltration profile as untreated mice at 4dpi with enrichment in inflammatory macrophages and monocytes (Fig S5H,I). These findings highlight clear efficacy of mAb treatment in controlling viral infection and viral titers but they underline the need for early treatment particularly in controlling the immunopathology, as has been noted clinically^22,53,54^

### Accurate timing of corticosteroids is necessary to balance viral clearance and prevent immunopathology.

**Figure 6.**
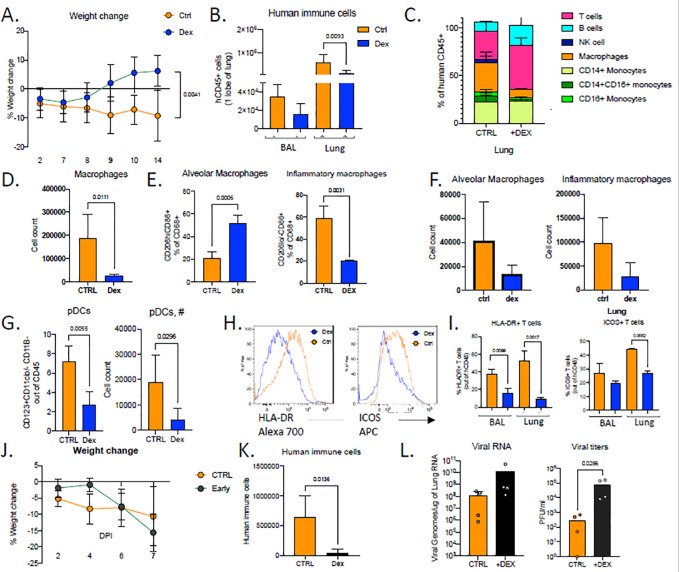
Accurate timing of corticosteroids is necessary to balance viral clearance and prevent immunopathology. **A.** Weight change in dexamethasone treated or control mice during SARS-Cov2 infection plotted as percent change compared with original weight prior to viral inoculation. Mice were treated with dexamethasone at 7,8,9 dpi. N=6–8. Unpaired, two-tailed t-test. **B.** Human immune cells in 14dpi lungs and BAL of MISTRG6-hACE2 mice treated with dexamethasone at 7, 8 9 dpi or left untreated. **C.** Human immune lineages lungs of dexamethasone treated or untreated mice within the human CD45+ population. Classical monocytes (CD14+), Intermediate monocytes (CD14+CD16), non-classical monocytes (CD16+CD14−), macrophages (CD68+), NK cells (NKP46+), T cells (CD3+), B cells (CD19+ and/or CD20+). MISTRG6-hACE2 mice were treated with dexamethasone at 7,8,9dpi. **D.** Number of human macrophages in lungs of dexamethasone treated and untreated mice at 14dpi. **E.** Frequencies of alveolar or inflammatory macrophages in the lungs of MISTRG6-hACE2 mice that were treated with dexamethasone or left untreated. **F.** Numbers of alveolar or inflammatory macrophages in the lungs of MISTRG6-hACE2 mice that were treated with dexamethasone or left untreated. **G.** Frequencies and numbers of pDCs at 14dpi in the lungs of dexamethasone treated or control mice. **H.** Representative plots for HLA-DR expression in lung T cells at 14dpi in dexamethasone treated or control mice. **I.** Frequencies of HLA-DR+ or ICOS+ T cells at 14dpi in the lungs and BAL of dexamethasone treated or control mice **J.** Weight change in dexamethasone treated or control mice during SARS-Cov2 infection plotted as percent change compared with original weight prior to viral inoculation. Mice were treated with dexamethasone at 3,4,5dpi. N=4–6. **K.** Human immune cells in lungs of MISTRG6-hACE2 mice treated with dexamethasone at 3, 4 5 dpi or left untreated (ctrl). N=4–6. Unpaired, two-tailed t-test. **L.** Viral RNA and viral titers measured by PFU in homogenized lung tissue at 7 dpi in dexamethasone treated or control mice. N=4–6. Mann-Whitney test, two-tailed. MISTRG6 mice were engrafted with CD34+ cells neonatally isolated from at least 2 donors. Pooled or infection matched representative results of at least 2 independent experiments are presented. Only P-values <0.05 are plotted. Mean with SD or individual values are plotted.

Our transcriptome analysis revealed glucocorticoids as possible upstream regulators of DEGs that are induced in infected lungs (Fig 4A–B). Moreover, given that dexamethasone has been so far the only therapeutic treatment that has impacted recovery and reduced mortality in a major way when given in patients with severe disease ^56^, we hypothesized that dexamethasone treatment in humanized mice may favorably impact immunopathology in mice infected with SARS-CoV-2. To test this hypothesis, we treated mice with dexamethasone for 3 days starting at 7dpi once the immune infiltration is established but viral titers were significantly reduced in the lungs (Fig. S6A). Indeed, mice treated with dexamethasone close to viral clearance (7dpi), recovered rapidly in weight by 14dpi and returned to weight gain comparable to their uninfected counterparts (Fig. 6A,1D). Dexamethasone treatment reduced human immune infiltrate and reversed many aspects of immune-activation (Fig. 6B). Mouse neutrophils in BAL were fewer in dexamethasone treated mice (Fig S6B). Macrophages, in particular inflammatory macrophages, were largely absent in the lungs of dexamethasone treated mice (Fig 6C–F). Dexamethasone treatment also blocked accumulation of pDCs and reduced T cell activation in lungs at both 14dpi and 28dpi (Fig 6J–L and S6E). Interestingly, dexamethasone treatment also blocked IgG specific B cell response as IgG+ B cells but not IgM+ B (Fig S6C). Relative contrubitions of these B cells to immune-pathology vs immune-protection remain open questions. It was notable that lack of immune cells in dexamethasone treated lungs also correlated with lowered viral RNA levels by 28dpi (Fig S6G). Given that the immune infiltrate is established early in mice (by 4dpi), we were prompted to investigate the timing of dexamethasone mediated control of immunopathology for COVID19. We therefore treated mice with dexamethasone for 3 days starting at 3dpi once the immune infiltration is established. In stark contrast to late dexamethasone treatment, early dexamethasone treated mice became moribund by 7dpi with rapidly declining weights compared with untreated mice (Fig. 6F). Dexamethasone-treated mice had significantly fewer immune cells infiltrating the lungs and in particular lacked inflammatory macrophages (Fig. 6K, S6G). Importantly, the disabled antiviral response in these mice led to significantly higher viral load in the lungs (Fig. 6L). These findings upon early dexamethasone treatment highlight the importance of the early antiviral response to contain viral infection treatment. The timing of dexamethasone treatments was instrumental in showcasing the necessity of the early anti-viral response in containing infection and the role of immune cells later in disease pathology.

## Discussion:

Accurate model systems that rapidly and comprehensively characterize COVID19 are and should remain pivotal in the development of promising discoveries. Here, we reveal a humanized mouse COVID-19 model that combines vector-based delivery of human ACE2 and a comprehensive human immune system that recapitulated both the innate and adaptive human immune systems during infection with SARS-CoV-2. Our unique model allowed for the first time chronic disease with SARS-CoV-2 infection in the context of patient-derived antibodies and characterized the potential players for immunopathology (Table S4), in particular the aberrant macrophage response that is thought to be the effector of disease morbidity and ARDS in patients^57^.

Our findings document that gross disease parameters such as weight loss and viral load were driven by the human immune system in our model, which suggested that human immune cells contribute uniquely to the pathology of human SARS-CoV2 infection. Sustained viral RNA and gross clinical features including failure to recover body weight even at very late time points post infection,are unique among animal models to our MISTRG6 humanized mice, with human immune cells and human ACE2 expression. Chronic disease manifestations were reflected in histopathological assessment of infected humanized lungs late in infection (14dpi and 28dpi). Significant cellular infiltrates, thickened septa and collagen deposition in lungs at 28dpi point to lack of recovery and fibrosis in infected humanized lungs long after infectious virus is cleared, recapitulating what is observed in severe human COVID19. To our knowledge, this is the only disease model that recapitulates chronic weight loss, sustained high viral RNA and chronic histopathology with pulmonary fibrosis seen in human patients^4,29–31,58^ and has not been observed in any of the prior animal (mice, ferrets, hamsters) models of COVID19 (Table S4)^18,25–28^. Nonetheless,chronic, humanized COVID-19 is not a lethal disease, which will make interesting further investigation of variables such as age, pre-existing health conditions and co-morbidities that contribute to high case fatality rate in humans.

Systemic T cell lymphopenia and sustained IFN response were other features of humanized COVID19 that mimic the human disease ^35,36,40^.^35,36,40^. Systemic, cyclical lymphopenia that follows rapid, strong activation of T cells in the infected lungs suggest that T cells are continuously recruited, activated and consumed in the lungs. Frequencies of splenic T cells negatively correlated with human genes such as *HIF1A, UBAP2L, MIF, FABP7* in lungs suggesting inflammation and stress response in lungs, possibly mediated by lung macrophages, may impact systemic lymphopenia. Genes that correlated with systemic lymphopenia were also enriched for interferon responsive genes (30/50 top correlating human genes and 26/50 top correlating mouse genes),and suggest some potential therapeutic targets to improve lymphopenia in patients.

We created a humanized mouse model of COVID19 that recapitulates the distribution and function of the human innate and adaptive immune system amenable to the mechanistic study of COVID 19 and its myriad of complications(Table S4). ***MISTRG6-hACE2*** mice could be used to study two aspects of the immune response, infectious viral clearance and immunopathology recapitulated in our model. We first evaluated patient-derived human antibodies to study this separation. Although prophylactic use of mAbs and to some extent early use of therapeutic use of mAbs attenuate disease parameters, systemic lymphopenia and immune infiltrates, late therapeutic use of mAbs or prophylactic use of convalescent plasma showed limited benefit similar to the human patient experience^22,53,54^, suggesting that the players of immune-pathology such as the inflammatory macrophages are seeded very early in infection. Despite efficient viral clearance, as measured by dramatically reduced viral titers and lack of antibody mediated enhancement of disease, mAbs when given late (36 hpi) did not help to prevent the potentially pathological inflammatory response. Our preclinical data are similar to the clinical experience where there are currently more than 20 SARS-CoV-2 specific monoclonal antidbodies in different stages of clinical trial testing. Results so far also support a reduction in viral load upon convalescent or monoclonal human antibody treatment, but clinical benefits are limited^53^. Our humanized model of COVID-19 could be particularly useful in evaluating efficacy and timing for these antibodies. When administred in a timely manner mAbs could be particularly useful in protecting uninfected individuals and preventing tranmission from an infected person by rapid clearance of infectious virus.

Separation of the two aspects of the immune response, infectious viral clearance and immunopathology recapitulated in our model, may prove to be useful in the context of controlling COVID19. Patients may benefit from early mAb treatment coupled with immune-suppression such as dexamethasone later in infection. Our data suggest that glucocorticoids, specifically dexamethasone, (which are decoded as upstream regulators of the inflammatory gene signature identified in humanized COVID19) should be considered only when viral titers are undetectable suggesting timing is crucial in promoting immune-protection while preventing pathology. highlighting the importance of accurate and frequent viral detection methods. When applied in the chronic time window dexamethasone very effectively controls immunopathology and reverses morbidity caused by COVID19. As expected, dexamethasone treatment has broad effects and reverses many aspects of immune-activation: macrophages, neutrophils, pDCs, T cells and B cells. Yet, non-specific suppression of the immune system using dexamethasone early in infection was catastrophic.

Our humanized mouse model of COVID19 is uniquely adapted to reflect patient heterogeneity but also provides consistency in a highly reproducible mouse model. Transcriptome analysis revealed differences between individual animals in the strength of the inflammatory response, which may in part help explain the variable outcome observed in disease morbidity, and mortality in human SARS-CoV-2 infection^59^. Yet, regardless of such heterogeneity, sustained interferon response, as has been postulated in humans^30,33,44^, was a common theme that shaped both the early anti-viral innate response as well as the late adaptive immune response in humanized COVID-19.

Emerging patient data detail more debilitating effects of COVID-19 in certain patient groups even in absence of previously described high risk criteria (age, pre-existing health conditions etc.). Although socio-economic factors are likely responsible for some or perhaps all of these effects, perturbation of our system should allow testing of the genuinely medical effects. Our humanized mouse system can be completely personalized by matching patient HSPCs with antibodies and medical history, allowing researchers to test novel therapeutics and other immunomodulatory agents to address conflicting reports in pre-clinical models and to predict efficacy in patients.

## Materials and Methods Mice

MISTRG6 was generated by the R. Flavell laboratory by combining mice generated by this lab, the laboratory of Markus Manz and Regeneron Pharmaceuticals based on the *Rag2−/− IL2rg−/−*129xBalb/c background supplemented with genes for human M-CSF, IL-3, SIRPα, thrombopoietin, GM-CSF and IL6 knocked into their respective mouse loci ^11,17^. MISTRG6 mice are deposited in Jackson Laboratories and made available to academic, non-profit and governmental institutions under a Yale-Regeneron material transfer agreement (already approved and agreed to by all parties). Instructions on obtaining the material transfer agreement for this mouse strain will be available along with strain information and upon request. All mice were maintained under specific pathogen free conditions in our animal facilities (either Biosafety Level 1,2 or 3) under our Animal Studies Committee-approved protocol. Unconstituted MISTRG6 mice were maintained with cycling treatment with enrofloxacin in the drinking water (Baytril, 0.27 mg/ml). All animal experimentations were performed in compliance with Yale Institutional Animal Care and Use Committee protocols. For SARS- CoV-2–infected mice, all procedures were performed in a BSL-3 facility with approval from the Yale Institutional Animal Care and Use Committee and Yale Environmental Health and Safety.

### AAV-hACE2 administration

AAV9 encoding hACE2 was purchased from Vector Biolabs (AAV9-CMV-hACE2). Animals were anaesthetized using isoflurane. The rostral neck was shaved and disinfected. A 5-mm incision was made, and the trachea was visualized. Using a 32-G insulin syringe, a 50-μl injection dose of 10^11^ genomic copies per milliliter of AAV-CMV-hACE2 was injected into the trachea. The incision was closed with 4–0 Vicryl suture and/or 3M Vetbond tissue adhesive. Following administration of analgesic animals were placed in a heated cage until full recovery. Mice were then moved to BSL-3 facilities for acclimation.

### SARS-CoV-2 infection

Mice were anesthetized using 20% vol/vol isoflurane diluted in propylene glycol. Using a pipette, 50 μl of SARS-CoV-2 (1–3×10^6^ PFU) was delivered intranasally.

### Viral titers

Mice were euthanized in 100% isoflurane. Approximately half of the right lung lobe was placed in a bead homogenizer tube with 1 ml of PBS + 2% FBS. After homogenization, 300 μl of this mixture was placed in 1mL Trizol (Invitrogen) for RNA extraction and analysis. Remaining volume of lung homogenates was cleared of debris by centrifugation (3,900 g for 10 min). Infectious titers of SARS-CoV-2 were determined by plaque assay in Vero E6 cells in DMEM 4% FBS, and 0.6% Avicel RC-581^60^. Plaques were resolved at 48 h after infection by fixing in 10% formaldehyde for 1 hour followed by staining for 1 hour in 0.5% crystal violet in 20% ethanol. Plates were rinsed in water to visualize plaques. Multiple dilutions of lung homegantes were used to quantify Infectious titers (minimum number of plaques that can be quantified= 10 per ml of lung homogenate)

### Viral RNA analysis

RNA was extracted with the RNeasy mini kit (Qiagen) per the manufacturer’s protocol. SARS-CoV-2 RNA levels were quantified using the Luna Universal Probe Onestep RT-qPCR kit (New England Biolabs) and US CDC real-time RT-PCR primer/probe sets for 2019-nCoV_N1. For each sample, 1 ug of RNA was used.

### Transplantation of human CD34+ hematopoietic progenitor cells into mice.

Fetal liver samples were cut in small fragments, treated for 45 min at 37 °C with collagenase D (Roche, 200 μg/ml), and prepared into a cell suspension. Human CD34+ cells were purified by performing density gradient centrifugation (Lymphocyte Separation Medium, MP Biomedicals), followed by positive immunomagnetic selection with EasySep ™ Human CD34 Positive Selection Kit (Stemcell). For intra-hepatic engraftment, newborn 1–3 day-old pups were injected with 20,000 fetal liver CD34+ cells in 20 μl of PBS were injected into the liver with a 22-gauge needle (Hamilton Company). All use of human materials was approved by the Yale University Human Investigation Committee.

### Isolation of cells and flow cytometry

All mice were analyzed at approximately 9–11 weeks of age. Single cell suspensions were prepared from blood, spleen BAL and lung. Mice were euthanized with 100% isoflurane. BAL was performed using standard methods with a 22G Catheter (BD). Blood was collected either retro-orbitally or via cardiac puncture following euthanasia. BAL was performed using standard methods with a 22G Catheter (BD)^61^. Lungs were harvested, minced and incubated in a digestion cocktail containing 1 mg/ml collagenase D (Sigma) and 30 μg/ml DNase I (Sigma-Aldrich) in RPMI at 37°C for 20 min. Tissue was then filtered through a 70-μm filter. Cells were treated with ammonium- chloride-potassium buffer and resuspended in PBS with 1*%* FBS. Mononuclear cells were incubated on ice with human (BD) and mouse (BioxCell, BE0307) Fc block for 10 min. After washing, primary antibody staining was performed at 4C for 20 min. After washing with PBS, cells were fixed using 4% paraformaldehyde. For intracellular staining, cells were washed with BD permeabilization buffer and stained in the same buffer for 45 min at room temperature. Samples were analyzed on an LSRII flow cytometer (BDBiosciences). Data were analyzed using FlowJo software.

### Antibodies

Antibodies against the following antigens were used:

Mouse antigens: CD45 (Clone: 30-F11), Ly6G (1A8), Ly6C (HK1.4), CD31(MEC13.3),CD326 (G8.8); Human antigens: CD45 (HI30), CD3 (UCHT1), CD14 (HCD14), CD16 (3G8), CD19 (HIB19), CD33 (WM53), CD20 (2H7), CD206 (15–2), CD86 (BU63), CD123(6H6), IGM (MHM-88), IGG(M1310G05) CD163 (GHI/61), CD169(7–239) CD68(Y1/82A), CD11B (M1/70), CD11C (3.9), HLA-DR(LN3), CD183 (G025H7), ICOS(C398.4A), PD1 (A17188B), NKp46 (9E2), CD56(MEM-188), CD4(OKT4), CD8(SK1), TCRGD(B1). All antibodies were obtained from Biolegend, unless otherwise specified. Convalescent plasma and monoclonal antibodies (clone 135 and 144) were acquired from M. Nussenzweig as has been previously described^23^.

### Bulk whole tissue lung RNA-sequencing

RNA isolated from homogenized lung tissue used for viral RNA analysis was also used for whole tissue transcriptome analysis. Libraries were made with the help of the Yale Center for Genomic Analysis. Briefly, libraries were prepared with an Illumina rRNA depletion kit and sequenced on a NovaSeq. Raw sequencing reads were aligned to the human-mouse combined genome with STAR [citation: https://doi.org/10.1093/bioinformatics/bts635], annotated and counted with HTSeq [citation: https://doi.org/10.1093/bioinformatics/btu638], normalized using DESeq2 [citation: https://doi.org/10.1186/s13059–014-0550–8], and graphed using the Broad Institute Morpheus web tool. Differential expression analysis was also performed with DESeq2. For IFN-stimulated gene identification, http://www.interferome.org was used with parameters -In Vivo, -Mus musculus or Homo sapiens -fold change up 2 and down 2.

### Single Cell RNA Sequencing 10X Genomics

Single cell suspensions from digested lungs were processed for droplet based scRNA-seq and 10000 cells were encapsulated into droplets using 10X Chromium GEM technololgy. Libraries were prepared in house using Chromium Next GEM Single Cell 3’ Reagent Kits v3.1 (10X Genomics). scRNA-seq libraries were sequenced using Nova-Seq. Raw sequencing reads were processed with Cell Ranger 3.1.0 using a human-mouse combined reference to generate a gene cell count matrix. To distinguish human and mouse cells, we counted the number of human genes (nHuman) and mouse genes (nMouse) with nonzero expression in each cell, and selected cells with nHuman > 20 * nMouse as human cells. The count matrix of human cells and human genes was used in the downstream analysis with Seurat 3.2 [citation: https://doi.org/10.1016/j.cell.2019.05.031]. Specifically, this matrix was filtered retaining cells with > 200 and < 5,000 genes and < 20% mitochondria transcripts. We then log transformed each entry of the matrix by computing log (CPM/100 + 1), where CPM stands for counts per million. To visualize the cell subpopulations in two dimensions, we applied principal component analysis followed by t-SNE, a nonlinear dimension reduction method, to the log-transformed data. Graph-based clustering was then used to generate clusters that were overlaid on the t-SNE coordinates to investigate cell subpopulations. Marker genes for each cluster of cells were identified using the Wilcoxon test with Seurat. For the adjusted P values the Bonferroni correction was used.

## Supplementary Material

Supplement **Table S3. Distribution of Type I interferon responsive genes in SARS-CoV-2 infected lungs during the course of infection.** Normalized counts for Type I interferon responsive genes within human DEGs are presented. For IFN-stimulated gene identification, http://www.interferome.org was used with parameters -In Vivo, Homo sapiens -fold change up 2 and down 2.

Supplement **Table S1. Differentiyally regulated genes (DEGs) in** SARS-CoV-2 **infected lungs.** Normalized counts, foldchanges in infected lungs compared with respect to uninfected mice and adjusted p values are presented for 516 mouse and 285 human differentially regulated genes.

Supplement Table S4: Comparison of COVID19 parameters and response to therapeutics in human patients, humanized mice and other animal models.

Supplement **Table S2: Pathway analysis of mouse and human DEGs in SARS-CoV-2 infected lungs** utilizing Gene Ontology(GO), Gene Set Enricment Analysis (GSEA) and Ingenuity pathway analysis platforms.

Supplement Figure S1 (matched to Figure 1):
A. Histopathology by H&E staining of infected lungs at higher magnifications at 28dpi. At least 4 mice per time point were analyzed.
Figure S2 (matched to Figure 2):
Representative gating strategy of human immune cells in the lungs of infected MISTRG-hACE2 mice.
Figure S3 (matched to Figure 3):
A. Frequencies of human CD4+ or CD8+ T cells within hCD3+ population in the blood and spleens of uninfected or infected mice at 2,4,7,14,28 dpi.
B. Numbers of human T cells in the blood pre and post-infection (2,4,7,14,28dpi).
C. Numbers of human T cells in the spleens of uninfected or infected mice (2,4,7,14,28dpi).
D. Frequencies of human CD4+ or CD8+ T cells within hCD3+ population in the lungs of uninfected or infected mice at 2,4,7,14,28 dpi.
E. IgG levels measured by ELISA in serum of uninfected or infected mice at 7,14,28 dpi.
F. CD11C and CD19 expression on human immune cells from infected lungs at 14 and 28 dpi.
G. Frequencies of human B cells marked by CD19 and CD20 expression within hCD45+ population in spleen and blood of uninfected or infected mice at 2,4,7,14,28 dpi.
H. Representative flow cytometry plots and frequencies of IgG+ B cells in the BAL of uninfected and infected mice 14 and 28dpi.
Mean with SD or individual values are plotted.
Figure S4 (matched to Figure 4):
A. Heatmap of normalized counts for bystander activated memory T cell signature genes (based on (Low et al., JEM 2020)) in lungs of MISTRG6-hACE2 mice infected with SARS-CoV-2.
B. Volcano plots showing foldchange and p values of differentially regulated genes at 28dpi compared to uninfected lungs. Genes with FC(Log2)>1 and p value<0.05 are highlighted in red. Replicates of at least 2 mice.
C. Heatmap of normalized counts for genes that are induced in B cells of patients with moderate or severe COVID19 in comparison with healthy controls. Normalized counts in lungs of uninfected or infected MISTRG6-hACE2 were plotted over the course of infection.
D. Heatmap of normalized counts for bystander activated memory B cell signature genes (based on Horns et al., 2020) )in lungs of MISTRG6-hACE2 mice infected with SARS-CoV-2.
Figure S5 (matched to Figure 5):
A. Schematic of experimental design of prophylactic antibody treatment MISTRG6-hACE2 mice received prophylactic treatment of convalescent plasma (5ml/kg) or monoclonal antibodies at 10mg/kg (clone 135-m135 or clone 144-m144 8 hours prior to infection or left untreated (untd). Mice were euthanized 4dpi.
B. Mean weight change in convalescent plasma treated mice at 2days and 4days post-infection plotted as percent change compared with original weight measured just before inoculation with SARS-Cov2.
C. Frequencies of human monocytes (CD14+ classical; CD14+CD16+ intermediate, CD16+ non classical) within human CD45+ cells in the lungs of MISTRG6-hACE2 mice which received a prophylactic treatment of monoclonal antibody clone 135 (m135) or clone 144(m144) 8 hours prior to infection or left untreated (untd). Unpaired, two-tailed t-test. P-values<0.05 are plotted. N=5–6.
D. Frequencies of human T cells within human CD45+ cells in spleens of MISTRG6-hACE2 mice received a prophylactic treatment of monoclonal antibody clone 135 (m135) or clone 144(m144) 8 hours prior to infection or left untreated (untd). Unpaired, two-tailed t-test. N=5–6.
E. Frequencies of human CD3+ T cells within human CD45+ population in the blood pre and post-infection (2,4,7,14,28dpi). Lines connect pre and post-infection values for the same mouse. MISTRG6-hACE2 mice received a prophylactic treatment of monoclonal antibody clone 135 (m135) or clone 144(m144) 8 hours prior to infection or left untreated (untd). Paired, two-tailed t-test. N=5–6. P-values<0.05 are plotted.
F. Schematic of experimental design of post-infection mAb treatment. MISTRG6-hACE2 mice received a mixed cocktail of monoclonal antibodies clone 135 (m135) and clone 144(m144) at 20mg/kg or left untreated (untd). Early treatment groups were treated 11hours post-infection and late treatment 35 hours post-infection.
G. Frequencies of human T cells within human CD45+ cells in spleens of MISTRG6-hACE2 mice that received early, late or no treatment of monoclonal antibody mix. Unpaired, two-tailed t-test. N=3–5.
H. Number of CD16+ human monocytes in lungs of treated and untreated mice at 4dpi. Mice were either MISTRG6-hACE2 mice that received a mixed cocktail of monoclonal antibodies clone 135 (m135) and clone 144(m144) or left untreated (untd). Early treatment groups was treated 11hours post-infection and late treatment 35 hours post-infection. Unpaired, two-tailed t-test. N=3–5.
I. Number of human macrophages in lungs of treated and untreated mice at 4dpi. MISTRG6-hACE2 mice that received a mixed cocktail of monoclonal antibodies clone 135 (m135) and clone 144(m144) or left untreated (untd). Early treatment group was treated 11hours post-infection and late treatment 35 hours post-infection. Unpaired, two-tailed t-test. N=3–5.
Pooled, infection matched representative results of at least 2 independent experiments are presented. P-values<0.05 are plotted. Mean with SD or individual values are plotted.
Figure S6 (matched to Figure 6):
A. Schematic of experimental design of SARS-Cov2 infected MISTRG6-hACE2 mice either treated with dexamethasone on days 7,8,9 dpi or left untreated.
B. Representative flow cytometry plots of Ly6G expressing SSChi cells within the mouse immune cell population (mouse CD45+) in the BAL of dexamethasone treated or control untreated mice.
C. HLA-DR expression on lung T cells 28dpi in dexamethasone treated or control mice.
D. Representative flow cytometry plots of Surface IgG and CD19 expression on human immune cells gated on hCD45+ cells in lungs of untreated or dexamethasone treated mice at 28dpi. N=4
E. Viral RNA in the lung homogenates of dexamethasone treated or control untreated mice at 28dpi. N=3–5. Mann-Whitney, two-tailed test.
F. Schematic of experimental design of SARS-Cov2 infected MISTRG6-hACE2 mice either treated with dexamethasone on days 3,4,5 dpi or left untreated.
F. Schematic of experimental design of SARS-Cov2 infected MISTRG6-hACE2 mice either treated with dexamethasone on days 3,4,5 dpi or left untreated.G. CD206 and CD68 expression in lung human immune cells in mice treated with dexamethasone or left untreated at 7dpi. CD206hi+ CD68+ cells are alveolar macrophages. N=4–6.

## Data Availability

All data that support the findings of this study are available within the paper and its Supplementary Information files. All 10x Genomics single cell RNA sequencing and bulk RNA sequencing data that support the findings of this study will be deposited in the Gene Expression Omnibus (GEO) repository with an accession code to be determined.

## References

[1] Letko M., Marzi A. & Munster V. Functional assessment of cell entry and receptor usage for SARS-CoV-2 and other lineage B betacoronaviruses. Nature microbiology 5, 562–569 (2020). 10.1038/s41564-020-0688-y PMC7095430 32094589

[2] Huang C. Clinical features of patients infected with 2019 novel coronavirus in Wuhan, China. The Lancet 395, 497–506, doi:10.1016/S0140-6736(20)30183-5 (2020). PMC7159299 31986264

[3] Mehta P. COVID-19: consider cytokine storm syndromes and immunosuppression. The Lancet (2020). 10.1016/S0140-6736(20)30628-0 PMC7270045 32192578

[4] Xu Z. Pathological findings of COVID-19 associated with acute respiratory distress syndrome. The Lancet. Respiratory medicine 8, 420–422, doi:10.1016/S2213-2600(20)30076-X (2020). 32085846 PMC7164771

[5] Theocharides A. P., Rongvaux A., Fritsch K., Flavell R. A. & Manz M. G. Humanized hemato-lymphoid system mice. Haematologica 101, 5–19, doi:10.3324/haematol.2014.115212 (2016). 26721800 PMC4697887

[6] Rongvaux A. Human hemato-lymphoid system mice: current use and future potential for medicine. Annu Rev Immunol 31, 635–674, doi:10.1146/annurev-immunol-032712-095921 (2013). 23330956 PMC4120191

[7] Rathinam C. Efficient differentiation and function of human macrophages in humanized CSF-1 mice. doi:10.1182/blood-2010-12-326926 (2011). 21791433

[8] Rongvaux A. Human thrombopoietin knockin mice efficiently support human hematopoiesis in vivo. Proceedings of the National Academy of Sciences of the United States of America 108, 2378–2383, doi:10.1073/pnas.1019524108 (2011). 21262827 PMC3038726

[9] Strowig T. Transgenic expression of human signal regulatory protein alpha in Rag2−/−gamma(c) −/−mice improves engraftment of human hematopoietic cells in humanized mice. Proceedings of the National Academy of Sciences of the United States of America 108, 13218–13223, doi:10.1073/pnas.1109769108 (2011). 21788509 PMC3156175

[10] Rongvaux A. Human thrombopoietin knockin mice efficiently support human hematopoiesis in vivo. Proceedings of the National Academy of Sciences of the United States of America 108, 2378–2383, doi:10.1073/pnas.1019524108 (2011). 21262827 PMC3038726

[11] Yu H. A novel humanized mouse model with significant improvement of class-switched, antigen-specific antibody production. Blood 129, 959–969, doi:10.1182/blood-2016-04-709584 (2017). 28077418 PMC5324713

[12] Rongvaux A. Development and function of human innate immune cells in a humanized mouse model. Nat Biotechnol 32, 364–372, doi:10.1038/nbt.2858 (2014). 24633240 PMC4017589

[13] Yu H. A novel humanized mouse model with significant improvement of class-switched, antigen-specific antibody production. Blood 129, 959–969, doi:10.1182/blood-2016-04-709584 (2017). 28077418 PMC5324713

[14] Billerbeck E. Development of human CD4+FoxP3+ regulatory T cells in human stem cell factor-, granulocyte-macrophage colony-stimulating factor-, and interleukin-3-expressing NOD-SCID IL2Rgamma(null) humanized mice. Blood 117, 3076–3086, doi:10.1182/blood-2010-08-301507 (2011). 21252091 PMC3062310

[15] Nicolini F. E., Cashman J. D., Hogge D. E., Humphries R. K. & Eaves C. J. NOD/SCID mice engineered to express human IL-3, GM-CSF and Steel factor constitutively mobilize engrafted human progenitors and compromise human stem cell regeneration. Leukemia 18, 341–347, doi:10.1038/sj.leu.2403222 (2004). 14628073

[16] Willinger T. Human IL-3/GM-CSF knock-in mice support human alveolar macrophage development and human immune responses in the lung. Proceedings of the National Academy of Sciences of the United States of America 108, 2390–2395, doi:10.1073/pnas.1019682108 (2011). 21262803 PMC3038773

[17] Rongvaux A. Development and function of human innate immune cells in a humanized mouse model. Nature Biotechnology 32, 364–372, doi:10.1038/nbt.2858 (2014). PMC4017589 24633240

[18] Israelow B. Mouse model of SARS-CoV-2 reveals inflammatory role of type I interferon signaling. J Exp Med 217, doi:10.1084/jem.20201241 (2020). PMC7401025 32750141

[19] Sippel T. R., Radtke S., Olsen T. M., Kiem H.-P. & Rongvaux A. Human hematopoietic stem cell maintenance and myeloid cell development in next-generation humanized mouse models. Blood advances 3, 268 (2019). 30696625 10.1182/bloodadvances.2018023887 PMC6373748

[20] Li W. Efficient replication of severe acute respiratory syndrome coronavirus in mouse cells is limited by murine angiotensin-converting enzyme 2. J Virol 78, 11429–11433, doi:10.1128/JVI.78.20.11429-11433.2004 (2004). 15452268 PMC521845

[21] Xu H. High expression of ACE2 receptor of 2019-nCoV on the epithelial cells of oral mucosa. International Journal of Oral Science 12, 8–8, doi:10.1038/s41368-020-0074-x (2020). 32094336 PMC7039956

[22] Chen P. SARS-CoV-2 neutralizing antibody LY-CoV555 in outpatients with Covid-19. New England Journal of Medicine (2020). 10.1056/NEJMoa2029849 PMC7646625 33113295

[23] Davide F. R. Convergent Antibody Responses to SARS-CoV-2 in Convalescent Individuals. Nature. 10.1038/s41586-020-2456-9 PMC7442695 32555388

[24] Hassan A. O. A SARS-CoV-2 infection model in mice demonstrates protection by neutralizing antibodies. Cell 182, 744–753. e744 (2020). 32553273 10.1016/j.cell.2020.06.011 PMC7284254

[25] Bao L. The pathogenicity of SARS-CoV-2 in hACE2 transgenic mice. Nature 583, 830–833, doi:10.1038/s41586-020-2312-y (2020). 32380511

[26] Roberts A. A mouse-adapted SARS-coronavirus causes disease and mortality in BALB/c mice. PLoS Pathog 3, e5, doi:10.1371/journal.ppat.0030005 (2007). 17222058 PMC1769406

[27] Sun S.-H. A mouse model of SARS-CoV-2 infection and pathogenesis. Cell Host & Microbe (2020). 10.1016/j.chom.2020.05.020 PMC7250783 32485164

[28] Rockx B. Comparative pathogenesis of COVID-19, MERS, and SARS in a nonhuman primate model. Science 368, 1012–1015 (2020). 32303590 10.1126/science.abb7314 PMC7164679

[29] Tian S. Pathological study of the 2019 novel coronavirus disease (COVID-19) through postmortem core biopsies. Modern Pathology, 1–8 (2020). 10.1038/s41379-020-0536-x PMC7156231 32291399

[30] Menter T. Postmortem examination of COVID-19 patients reveals diffuse alveolar damage with severe capillary congestion and variegated findings in lungs and other organs suggesting vascular dysfunction. Histopathology 77, 198–209 (2020). 32364264 10.1111/his.14134 PMC7496150

[31] Barton L. M., Duval E. J., Stroberg E., Ghosh S. & Mukhopadhyay S. Covid-19 autopsies, oklahoma, usa. American Journal of Clinical Pathology 153, 725–733 (2020). 32275742 10.1093/ajcp/aqaa062 PMC7184436

[32] Baharom F. Dendritic cells and monocytes with distinct inflammatory responses reside in lung mucosa of healthy humans. The Journal of Immunology 196, 4498–4509 (2016). 27183618 10.4049/jimmunol.1600071

[33] Zhou Z. Heightened Innate Immune Responses in the Respiratory Tract of COVID-19 Patients. 27, 883–890 e882, doi:10.1016/j.chom.2020.04.017 (2020). PMC7196896 32407669

[34] Liao M. Single-cell landscape of bronchoalveolar immune cells in patients with COVID-19. Nature medicine, 1–3 (2020). 10.1038/s41591-020-0901-9 32398875

[35] Mathew D. Deep immune profiling of COVID-19 patients reveals distinct immunotypes with therapeutic implications. Science 369 (2020). 10.1126/science.abc8511 PMC7402624 32669297

[36] Chen Z. & Wherry E. J. T cell responses in patients with COVID-19. Nature Reviews Immunology, 1–8 (2020). 10.1038/s41577-020-0402-6 PMC7389156 32728222

[37] Lucas C. Longitudinal analyses reveal immunological misfiring in severe COVID-19. Nature 584, 463–469 (2020). 32717743 10.1038/s41586-020-2588-y PMC7477538

[38] Wang S. IL-21 drives expansion and plasma cell differentiation of autoreactive CD11c hi T-bet+ B cells in SLE. Nature communications 9, 1–14 (2018). 10.1038/s41467-018-03750-7 PMC5931508 29717110

[39] Jenks S. A. Distinct effector B cells induced by unregulated toll-like receptor 7 contribute to pathogenic responses in systemic lupus erythematosus. Immunity 49, 725–739. e726 (2018). 30314758 10.1016/j.immuni.2018.08.015 PMC6217820

[40] Woodruff M. C. Extrafollicular B cell responses correlate with neutralizing antibodies and morbidity in COVID-19. Nature immunology 21, 1506–1516 (2020). 33028979 10.1038/s41590-020-00814-z PMC7739702

[41] Schultheiß C. Next-generation sequencing of T and B cell receptor repertoires from COVID-19 patients showed signatures associated with severity of disease. Immunity 53, 442–455. e444 (2020). 32668194 10.1016/j.immuni.2020.06.024 PMC7324317

[42] Stephens D. S. & McElrath M. J. COVID-19 and the Path to Immunity. Jama 324, 1279–1281 (2020). 32915201 10.1001/jama.2020.16656 PMC12177933

[43] Grant R. A. Circuits between infected macrophages and T cells in SARS-CoV-2 pneumonia. Nature, 1–10. 10.1038/s41586-020-03148-w PMC7987233 33429418

[44] Dolan M. E. Investigation of COVID-19 comorbidities reveals genes and pathways coincident with the SARS-CoV-2 viral disease. Scientific reports 10, 1–11 (2020). 33257774 10.1038/s41598-020-77632-8 PMC7704638

[45] Nienhold R. Two distinct immunopathological profiles in autopsy lungs of COVID-19. Nature communications 11, 1–13 (2020). 10.1038/s41467-020-18854-2 PMC7546638 33033248

[46] Major J. Type I and III interferons disrupt lung epithelial repair during recovery from viral infection. Science 369, 712–717, doi:10.1126/science.abc2061 (2020). 32527928 PMC7292500

[47] Del Valle D. M. An inflammatory cytokine signature predicts COVID-19 severity and survival. Nat Med 26, 1636–1643, doi:10.1038/s41591-020-1051-9 (2020). 32839624 PMC7869028

[48] Leisman D. E. Cytokine elevation in severe and critical COVID-19: a rapid systematic review, meta-analysis, and comparison with other inflammatory syndromes. The Lancet Respiratory Medicine (2020). 10.1016/S2213-2600(20)30404-5 PMC7567529 33075298

[49] Low J. S. Tissue-resident memory T cell reactivation by diverse antigen-presenting cells imparts distinct functional responses. Journal of Experimental Medicine 217 (2020). 10.1084/jem.20192291 PMC7398161 32525985

[50] Kaneko N. Loss of Bcl-6-expressing T follicular helper cells and germinal centers in COVID-19. Cell 183, 143–157. e113 (2020). 32877699 10.1016/j.cell.2020.08.025 PMC7437499

[51] Hutcheson J. Combined deficiency of proapoptotic regulators Bim and Fas results in the early onset of systemic autoimmunity. Immunity 28, 206–217 (2008). 18275831 10.1016/j.immuni.2007.12.015

[52] Horns F., Dekker C. L. & Quake S. R. Memory B cell activation, broad anti-influenza antibodies, and bystander activation revealed by single-cell transcriptomics. Cell Reports 30, 905–913. e906 (2020). 31968262 10.1016/j.celrep.2019.12.063 PMC7891556

[53] Cruz-Teran C. Challenges and opportunities for antiviral monoclonal antibodies as COVID-19 therapy. Advanced Drug Delivery Reviews (2020). 10.1016/j.addr.2020.12.004 PMC7833882 33309815

[54] Casadevall A. & Pirofski L.-a. The convalescent sera option for containing COVID-19. The Journal of clinical investigation 130 (2020). 10.1172/JCI138003 PMC7108922 32167489

[55] Schäfer A. Antibody potency, effector function, and combinations in protection and therapy for SARS-CoV-2 infection in vivo. Journal of Experimental Medicine 218 (2020). 10.1084/jem.20201993 PMC7673958 33211088

[56] Group R. C. Dexamethasone in hospitalized patients with Covid-19—preliminary report. New England Journal of Medicine (2020).

[57] Han S. & Mallampalli R. K. The acute respiratory distress syndrome: from mechanism to translation. The Journal of Immunology 194, 855–860 (2015). 25596299 10.4049/jimmunol.1402513 PMC4299926

[58] Bussani R. Persistence of viral RNA, pneumocyte syncytia and thrombosis are hallmarks of advanced COVID-19 pathology. EBioMedicine 61, 103104 (2020). 33158808 10.1016/j.ebiom.2020.103104 PMC7677597

[59] Ware L. B. Physiological and biological heterogeneity in COVID-19-associated acute respiratory distress syndrome. The Lancet Respiratory Medicine (2020). 10.1016/S2213-2600(20)30369-6 PMC7836300 32861277

[60] Wei J. Genome-wide CRISPR screens reveal host factors critical for SARS-CoV-2 infection. Cell (2020). 10.1016/j.cell.2020.10.028 PMC7574718 33147444

[61] Sun F., Xiao G. & Qu Z. Murine bronchoalveolar lavage. Bio-protocol 7, e2287 (2017). 29082285 10.21769/BioProtoc.2287 PMC5659634

