# Supplementary material for: A humanized mouse model of chronic COVID-19 to evaluate disease mechanisms and treatment options
Source: Res Sq. 2021 Mar 17:rs.3.rs-279341. Preprint. [Version 1] doi: 10.21203/rs.3.rs-279341/v1 (PMC7987100; doi:10.21203/rs.3.rs-279341/v1)
Supplement: Supplement — Table S4: Comparison of COVID19 parameters and response to therapeutics in human patients, humanized mice and other animal models. [file 6952aada398889fe35c7d9ee.docx]

Table 1: Comparison of COVID-19 (disease parameters, manifestations and therapeutics) in humans, humanized mice and other animal models

| Disease parameter | **Human COVID-19** | **Humanized COVID-19** | **COVID-19 in animal models** |
| --- | --- | --- | --- |
| Weight loss | >5% of initial body weight | Chronic  10-30% of initial body weight, sustained for weeks | Acute  Hamsters^1^: 10% weight loss, recovers in 10 days  Mouse adapted SARS-2 virus in mice^2,3^:10- 20 % weight loss, recovers in ~10 days |
| Duration of disease pathology | Weeks to months^4^ | >4 weeks | 4-10 days |
| Mortality | 0.1% to over 25% case fatality rate based on age, risk factors and country | None detected | ﻿Fatal encephalitis in mouse brain upon vector mediated, transient ACE2 expressing model^5^ |
| Lung pathology | Diffuse alveolar damage, perivenular, fibrosis in severe cases^6-9^ | Chronic  Diffuse alveolar damage, signs of fibrosis with significant cellular infiltrate late in disease | ﻿Acute  Vector-mediated or transgenic mouse models^10-12^: Mild alveolitis  Ferrets^13^: mostly upper respiratory disease with infiltration in alveolar spaces  Mouse adapted SARS-2 virus in mice^3^: Acute lung injury, resolves in days |
| Lymphopenia | T cell lymphopenia^14^ ^4,15^(CD8+ T cell in particular) | T cell lymphopenia (CD8+ T cell in particular) | None |
| Macrophage response | Monocytic, inflammatory macrophages as drivers of pathology^16,17^ | Monocytic, inflammatory macrophages as drivers of pathology | Monocytes and monocytic macrophages infiltrate the lungs but immunopathology resolves^10^ |
| Cytokines | Increased levels of  proinflammatory cytokines/chemokines : IL6, TNF, IL1B, IFNγ, MCP-1-CCL2, CXCL10 and others^14,16-18^ | Increased levels of  proinflammatory cytokines  (IL6, TNF, IL1B, IFNγ, MCP1 and others) | Increased levels of  proinflammatory cytokines  (IL6, TNF, IL1B, IFNγ, MCP1 and others) particularly in mouse adapted version of SARS-2^3,10^ |
| T cell response | Activated T cells with TCR diversity correlating with better prognosis^14,18,19^ | Activated T cells | Activated T cells |
| IFN signature | Sustained for weeks | Sustained for weeks | Sustained for days, responsible for recruitment of Macs^5^ |
| B cell response | Defect in germinal centers^20^; extrafollicular^21^, highly inflammatory^18,19^ | Extrafollicular, highly inflammatory | Germinal center response |
| Therapeutics  (Dexamethasone, Abs) | Dexamethasone^22^: reduced mortality  Convalescent and monoclonal Abs^23-25^: limited response, viral clearance | Dexamethasone: timing is crucial to limit immunopathology  Convalescent and monoclonal Abs: beneficial as prophylactics and after early administration, late administration provides no benefit, limited response, viral titers reduced or cleared at 4dpi in all applications | Transfer of neutralizing antibodies and mAb stop disease^12,13^. |
|  |  |  |  |

References:

1 Imai, M. *et al.* Syrian hamsters as a small animal model for SARS-CoV-2 infection and countermeasure development. *Proceedings of the National Academy of Sciences* **117**, 16587-16595 (2020).

2 Roberts, A. *et al.* A mouse-adapted SARS-coronavirus causes disease and mortality in BALB/c mice. *PLoS Pathog* **3**, e5, doi:10.1371/journal.ppat.0030005 (2007).

3 Dinnon, K. H. *et al.* A mouse-adapted model of SARS-CoV-2 to test COVID-19 countermeasures. *Nature* **586**, 560-566 (2020).

4 Huang, C. *et al.* Clinical features of patients infected with 2019 novel coronavirus in Wuhan, China. *The Lancet* **395**, 497-506, doi:10.1016/S0140-6736(20)30183-5 (2020).

5 Song, E. *et al.* Neuroinvasion of SARS-CoV-2 in human and mouse brain. *Journal of Experimental Medicine* **218** (2021).

6 Tian, S. *et al.* Pathological study of the 2019 novel coronavirus disease (COVID-19) through postmortem core biopsies. *Modern Pathology*, 1-8 (2020).

7 Menter, T. *et al.* Postmortem examination of COVID‐19 patients reveals diffuse alveolar damage with severe capillary congestion and variegated findings in lungs and other organs suggesting vascular dysfunction. *Histopathology* **77**, 198-209 (2020).

8 Barton, L. M., Duval, E. J., Stroberg, E., Ghosh, S. & Mukhopadhyay, S. Covid-19 autopsies, oklahoma, usa. *American Journal of Clinical Pathology* **153**, 725-733 (2020).

9 Xu, Z. *et al.* Pathological findings of COVID-19 associated with acute respiratory distress syndrome. *The Lancet. Respiratory medicine* **8**, 420-422, doi:10.1016/S2213-2600(20)30076-X (2020).

10 Israelow, B. *et al.* Mouse model of SARS-CoV-2 reveals inflammatory role of type I interferon signaling. *J Exp Med* **217**, doi:10.1084/jem.20201241 (2020).

11 Sun, S.-H. *et al.* A mouse model of SARS-CoV-2 infection and pathogenesis. *Cell Host & Microbe* (2020).

12 Hassan, A. O. *et al.* A SARS-CoV-2 infection model in mice demonstrates protection by neutralizing antibodies. *Cell* **182**, 744-753. e744 (2020).

13 Ter Meulen, J. *et al.* Human monoclonal antibody as prophylaxis for SARS coronavirus infection in ferrets. *The Lancet* **363**, 2139-2141 (2004).

14 Mathew, D. *et al.* Deep immune profiling of COVID-19 patients reveals distinct immunotypes with therapeutic implications. *Science* **369** (2020).

15 Tan, L. *et al.* Lymphopenia predicts disease severity of COVID-19: a descriptive and predictive study. *Signal transduction and targeted therapy* **5**, 1-3 (2020).

16 Zhang, J.-Y. *et al.* Single-cell landscape of immunological responses in patients with COVID-19. *Nature immunology* **21**, 1107-1118 (2020).

17 Liao, M. *et al.* Single-cell landscape of bronchoalveolar immune cells in patients with COVID-19. *Nature medicine*, 1-3 (2020).

18 Lucas, C. *et al.* Longitudinal analyses reveal immunological misfiring in severe COVID-19. *Nature* **584**, 463-469 (2020).

19 Chen, Z. & Wherry, E. J. T cell responses in patients with COVID-19. *Nature Reviews Immunology*, 1-8 (2020).

20 Kaneko, N. *et al.* Loss of Bcl-6-expressing T follicular helper cells and germinal centers in COVID-19. *Cell* **183**, 143-157. e113 (2020).

21 Woodruff, M. C. *et al.* Extrafollicular B cell responses correlate with neutralizing antibodies and morbidity in COVID-19. *Nature immunology* **21**, 1506-1516 (2020).

22 Group, R. C. Dexamethasone in hospitalized patients with Covid-19—preliminary report. *New England Journal of Medicine* (2020).

23 Cruz-Teran, C. *et al.* Challenges and opportunities for antiviral monoclonal antibodies as COVID-19 therapy. *Advanced Drug Delivery Reviews* (2020).

24 Casadevall, A. & Pirofski, L.-a. The convalescent sera option for containing COVID-19. *The Journal of clinical investigation* **130** (2020).

25 Chen, P. *et al.* SARS-CoV-2 neutralizing antibody LY-CoV555 in outpatients with Covid-19. *New England Journal of Medicine* (2020).
