# Supplementary material for: A humanized mouse model of chronic COVID-19 to evaluate disease mechanisms and treatment options
Source: Res Sq. 2021 Mar 17:rs.3.rs-279341. Preprint. [Version 1] doi: 10.21203/rs.3.rs-279341/v1 (PMC7987100; doi:10.21203/rs.3.rs-279341/v1)
Supplement: Supplement — Figure S1 (matched to Figure 1): A. Histopathology by H&E staining of infected lungs at higher magnifications at 28dpi. At least 4 mice per time point were analyzed. Figure S2 (matched to Figure 2): Representative gating strategy of human immune cells in the lungs of infected MISTRG-hACE2 mice. Figure S3 (matched to Figure 3): A. Frequencies of human CD4+ or CD8+ T cells within hCD3+ population in the blood and spleens of uninfected or infected mice at 2,4,7,14,28 dpi. B. Numbers of human T cells in the blood pre and post-infection (2,4,7,14,28dpi). C. Numbers of human T cells in the spleens of uninfected or infected mice (2,4,7,14,28dpi). D. Frequencies of human CD4+ or CD8+ T cells within hCD3+ population in the lungs of uninfected or infected mice at 2,4,7,14,28 dpi. E. IgG levels measured by ELISA in serum of uninfected or infected mice at 7,14,28 dpi. F. CD11C and CD19 expression on human immune cells from infected lungs at 14 and 28 dpi. G. Frequencies of human B cells marked by CD19 and CD20 expression within hCD45+ population in spleen and blood of uninfected or infected mice at 2,4,7,14,28 dpi. H. Representative flow cytometry plots and frequencies of IgG+ B cells in the BAL of uninfected and infected mice 14 and 28dpi. Mean with SD or individual values are plotted. Figure S4 (matched to Figure 4): A. Heatmap of normalized counts for bystander activated memory T cell signature genes (based on (Low et al., JEM 2020)) in lungs of MISTRG6-hACE2 mice infected with SARS-CoV-2. B. Volcano plots showing foldchange and p values of differentially regulated genes at 28dpi compared to uninfected lungs. Genes with FC(Log2)>1 and p value<0.05 are highlighted in red. Replicates of at least 2 mice. C. Heatmap of normalized counts for genes that are induced in B cells of patients with moderate or severe COVID19 in comparison with healthy controls. Normalized counts in lungs of uninfected or infected MISTRG6-hACE2 were plotted over the course of infection. D. Heatmap of nor [file a8351d397e318bf3cb96c982.pdf]

Figure S1

A.

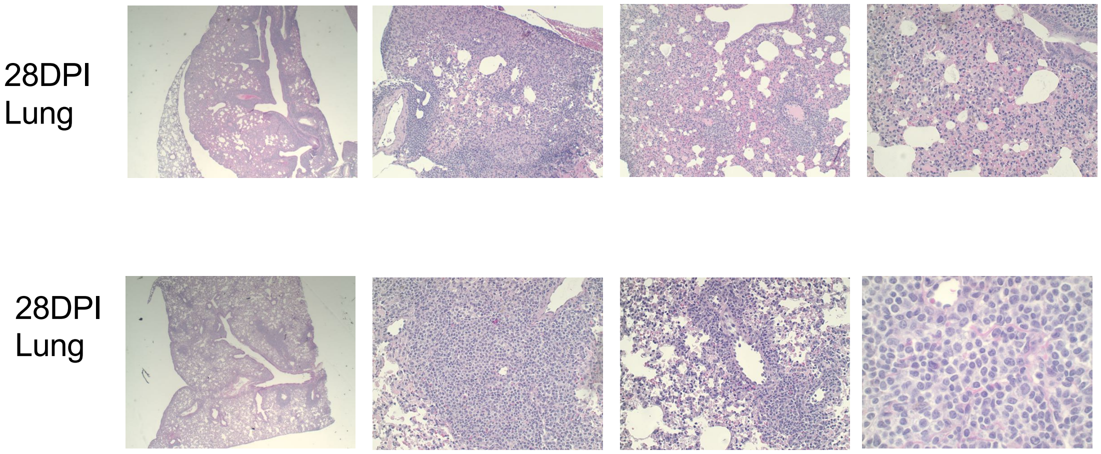

Figure S1:

A. Histopathology by H&E staining of infected lungs at higher magnifications at 28dpi. At least 4 mice per time point were analyzed.

Figure S2

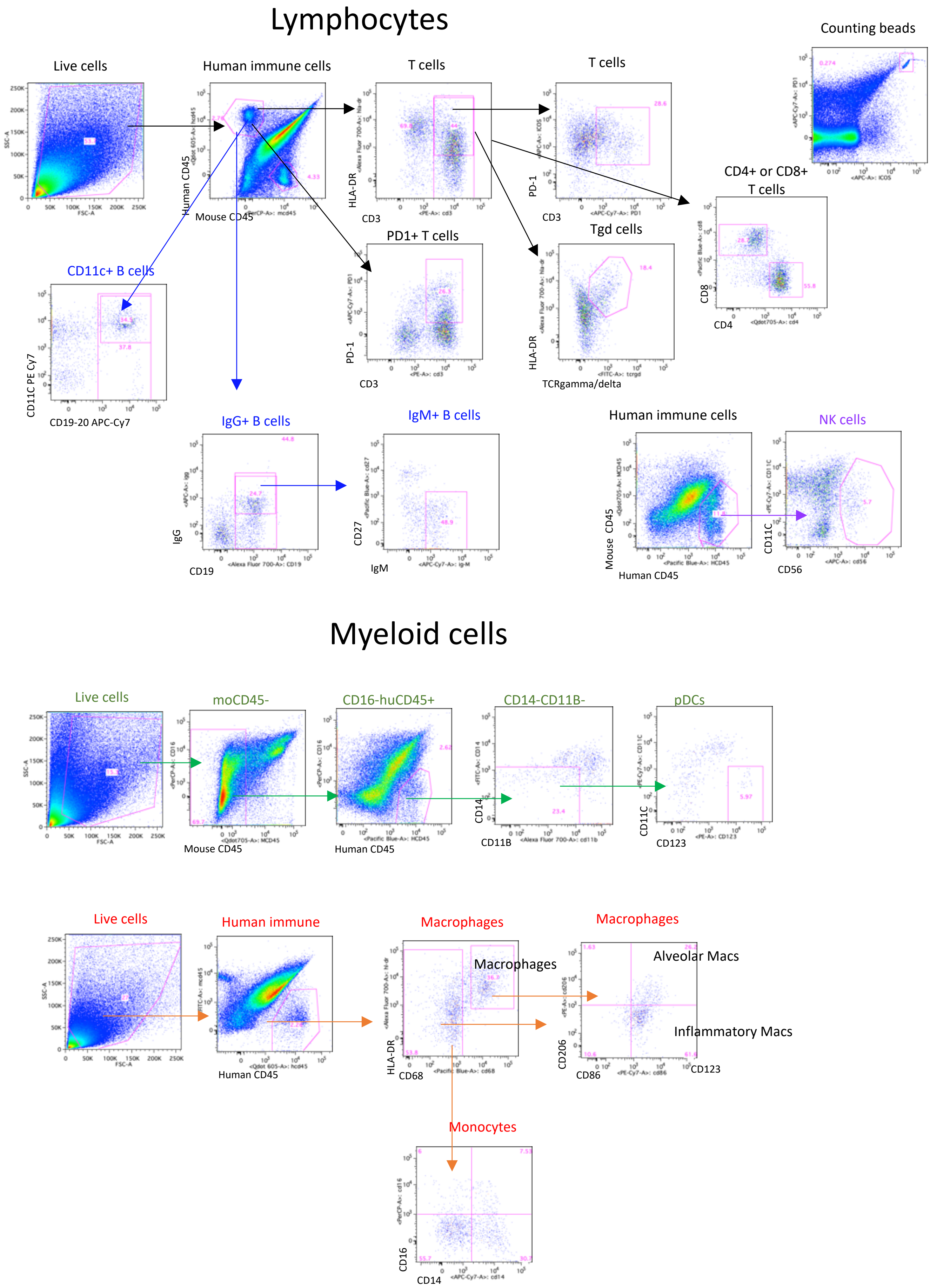

Figure S2:
Representative gating strategy of human immune cells in the lungs of infected MISTRG-hACE2 mice.

Figure S3

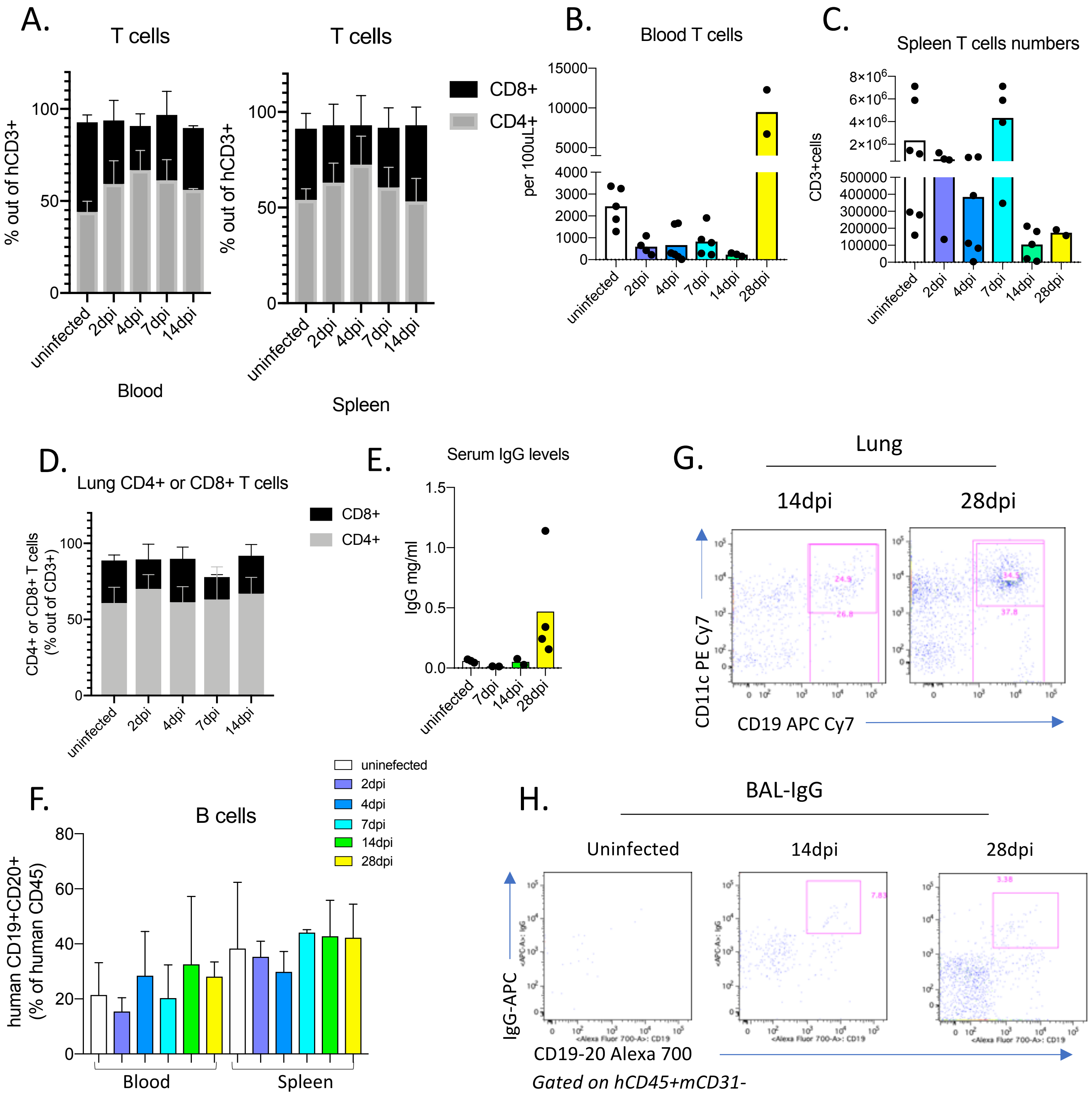

Figure S3.

A. Frequencies of human CD4+ or CD8+ T cells within hCD3+ population in the blood and spleens of uninfected or infected mice at 2,4,7,14,28 dpi.

B. Numbers of human T cells in the blood pre and post-infection (2,4,7,14,28dpi).

C. Numbers of human T cells in the spleens of uninfected or infected mice (2,4,7,14,28dpi).

D. Frequencies of human CD4+ or CD8+ T cells within hCD3+ population in the lungs of uninfected or infected mice at 2,4,7,14,28 dpi.

E. IgG levels measured by ELISA in serum of uninfected or infected mice at 7,14,28 dpi.

F. CD11C and CD19 expression on human immune cells from infected lungs at 14 and 28 dpi.

G. Frequencies of human B cells marked by CD19 and CD20 expression within hCD45+ population in spleen and blood of uninfected or infected mice at 2,4,7,14,28 dpi.

H. Representative flow cytometry plots and frequencies of IgG+ B cells in the BAL of uninfected and infected mice 14 and 28dpi.

Mean with SD or individual values are plotted.

Figure S4:

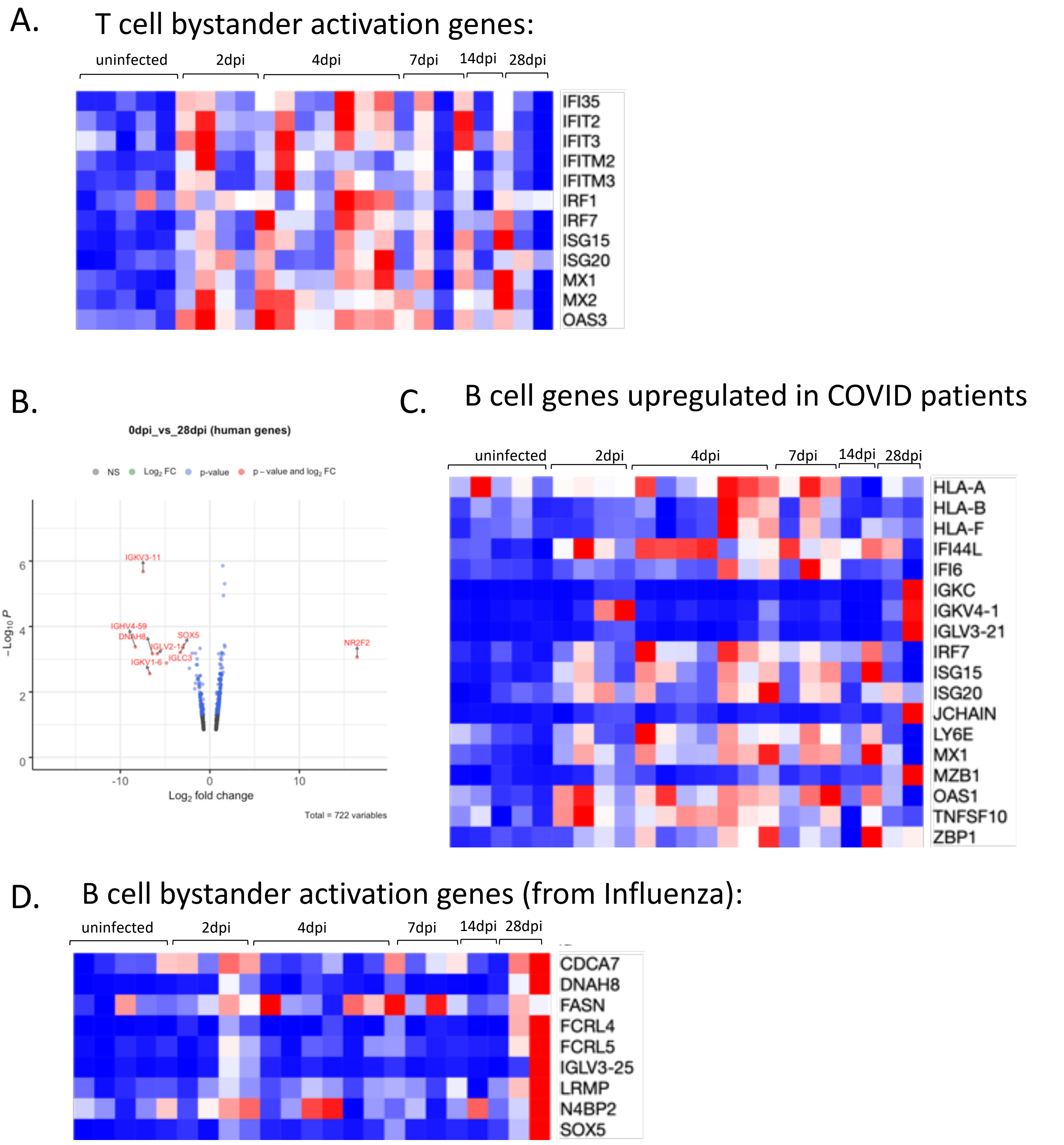

Figure S4.

A. Heatmap of normalized counts for bystander activated memory T cell signature genes (based on (Low et al., JEM 2020)) in lungs of MISTRG6-hACE2 mice infected with SARS-CoV-2.

B. Volcano plots showing foldchange and p values of differentially regulated genes at 28dpi compared to uninfected lungs. Genes with FC(Log2)>1 and p value<0.05 are highlighted in red. Replicates of at least 2.

C. Heatmap of normalized counts for genes that are induced in B cells of patients with moderate or severe COVID19 in comparison with healthy controls. Normalized counts in lungs of uninfected or infected MISTRG6-hACE2 were plotted over the course of infection.

D. Heatmap of normalized counts for bystander activated memory B cell signature genes (based on Horns et al., 2020))in lungs of MISTRG6-hACE2 mice infected with SARS-CoV-2.

Figure S5.

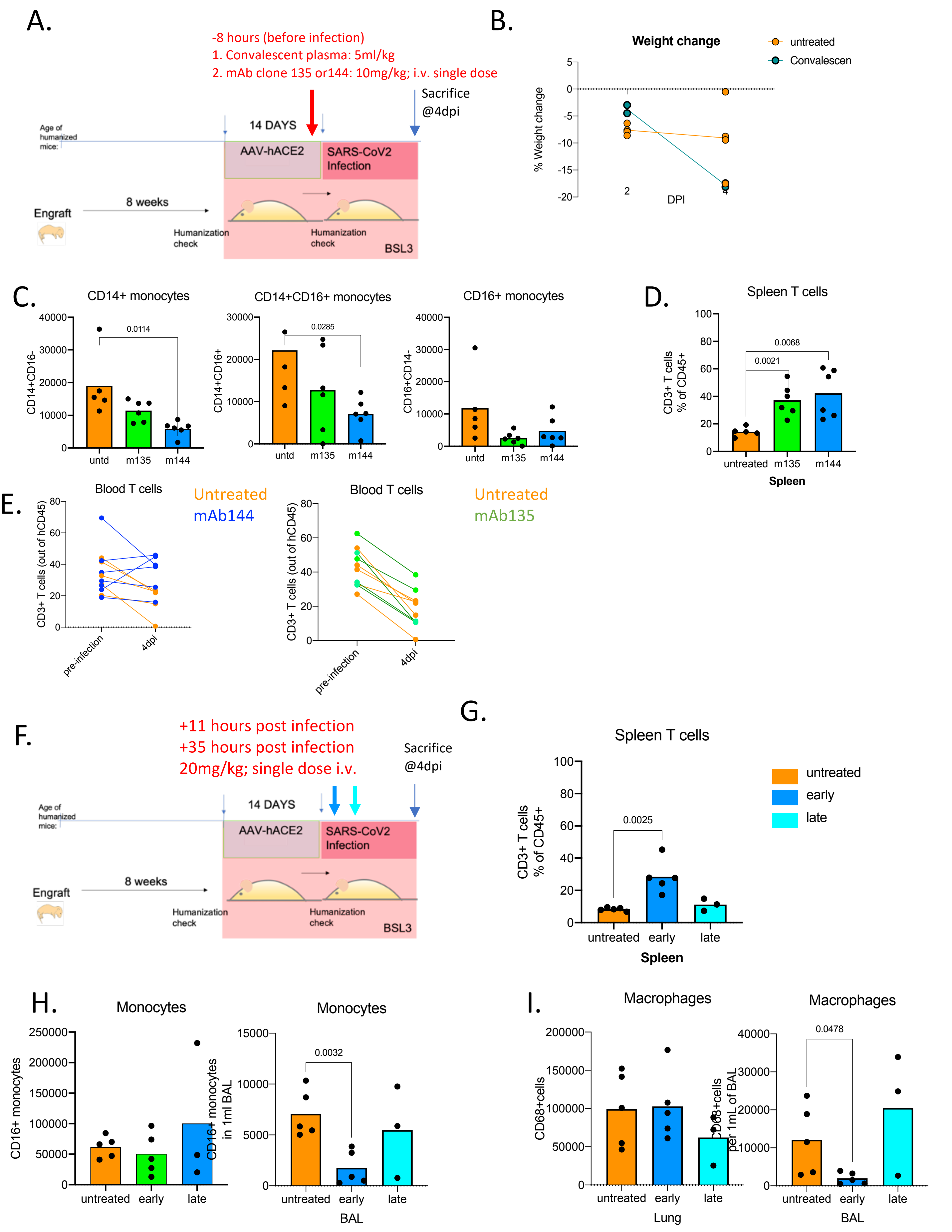
A.
-8 hours (before infection)
1. Convalescent plasma: 5ml/kg
2. mAb clone 135 or144: 10mg/kg; i.v. single dose
Sacrifice @4dpi
Age of humanized mice:
14 DAYS
AAV-hACE2
SARS-CoV2 Infection
8 weeks
Engraft
Humanization check
Humanization check
BSL3
B.
Weight change
untreated
Convalescen
% Weight change
DPI
C.
CD14+ monocytes
CD14+CD16-
0.0114
untd
m135
m144
CD14+CD16+ monocytes
CD14+CD16+
0.0285
CD16+ monocytes
CD16+CD14-
D.
Spleen T cells
CD3+ T cells % of CD45+
0.0068
0.0021
untreated
Spleen
E.
Blood T cells
Untreated
mAb144
CD3+ T cells (out of hCD45)
pre-infection
4dpi
Blood T cells
Untreated
mAb135
F.
+11 hours post infection
+35 hours post infection
20mg/kg; single dose i.v.
Sacrifice @4dpi
G.
Spleen T cells
untreated
early
late
0.0025
H.
Monocytes
CD16+ monocytes
CD16+ monocytes in 1ml BAL
0.0032
BAL
I.
Macrophages
CD68+cells
Lung
CD68+cells per 1mL of BAL
0.0478

Figure S5.

A. Schematic of experimental design of prophylactic antibody treatment MISTRG6-hACE2 mice received prophylactic treatment of convalescent plasma (5ml/kg) or monoclonal antibodies at 10mg/kg (clone 135 - m135 or clone 144-m144 8 hours prior to infection or left untreated (untd). Mice were euthanized 4dpi.
B. Mean weight change in convalescent plasma treated mice at 2days and 4days post-infection plotted as percent change compared with original weight measured just before inoculation with SARS-Cov2.
C. Frequencies of human monocytes (CD14+ classical; CD14+CD16+ intermediate, CD16+ non classical) within human CD45+ cells in the lungs of MISTRG6-hACE2 mice which received a prophylactic treatment of monoclonal antibody clone 135 (m135) or clone 144(m144) 8 hours prior to infection or left untreated (untd). Unpaired, two-tailed t-test. P-values<0.05 are plotted. N=5-6.
D. Frequencies of human T cells within human CD45+ cells in spleens of MISTRG6-hACE2 mice received a prophylactic treatment of monoclonal antibody clone 135 (m135) or clone 144(m144) 8 hours prior to infection or left untreated (untd). Unpaired, two-tailed t-test. N=5-6.
E. Frequencies of human CD3+ T cells within human CD45+ population in the blood pre and post-infection (2,4,7,14,28dpi). Lines connect pre and post-infection values for the same mouse. MISTRG6-hACE2 mice received a prophylactic treatment of monoclonal antibody clone 135 (m135) or clone 144(m144) 8 hours prior to infection or left untreated (untd). Paired, two-tailed t-test. N=5-6. P-values<0.05 are plotted.
F. Schematic of experimental design of post-infection mAb treatment. MISTRG6-hACE2 mice received a mixed cocktail of monoclonal antibodies clone 135 (m135) and clone 144(m144) at 20mg/kg or left untreated (untd). Early treatment groups were treated 11hours post-infection and late treatment 35 hours post-infection.
G. Frequencies of human T cells within human CD45+ cells in spleens of MISTRG6-hACE2 mice that received early, late or no treatment of monoclonal antibody mix. Unpaired, two-tailed t-test. N=3-5.
H. Number of CD16+ human monocytes in lungs of treated and untreated mice at 4dpi. Mice were either MISTRG6-hACE2 mice that received a mixed cocktail of monoclonal antibodies clone 135 (m135) and clone 144(m144) or left untreated (untd). Early treatment groups was treated 11hours post-infection and late treatment 35 hours post-infection. Unpaired, two-tailed t-test. N=3-5.
I. Number of human macrophages in lungs of treated and untreated mice at 4dpi. MISTRG6-hACE2 mice that received a mixed cocktail of monoclonal antibodies clone 135 (m135) and clone 144(m144) or left untreated (untd). Early treatment group was treated 11hours post-infection and late treatment 35 hours post-infection. Unpaired, two-tailed t-test. N=3-5.

Pooled, infection matched representative results of at least 2 independent experiments are presented. P-values<0.05 are plotted. Mean with SD or individual values are plotted.

Figure S6.

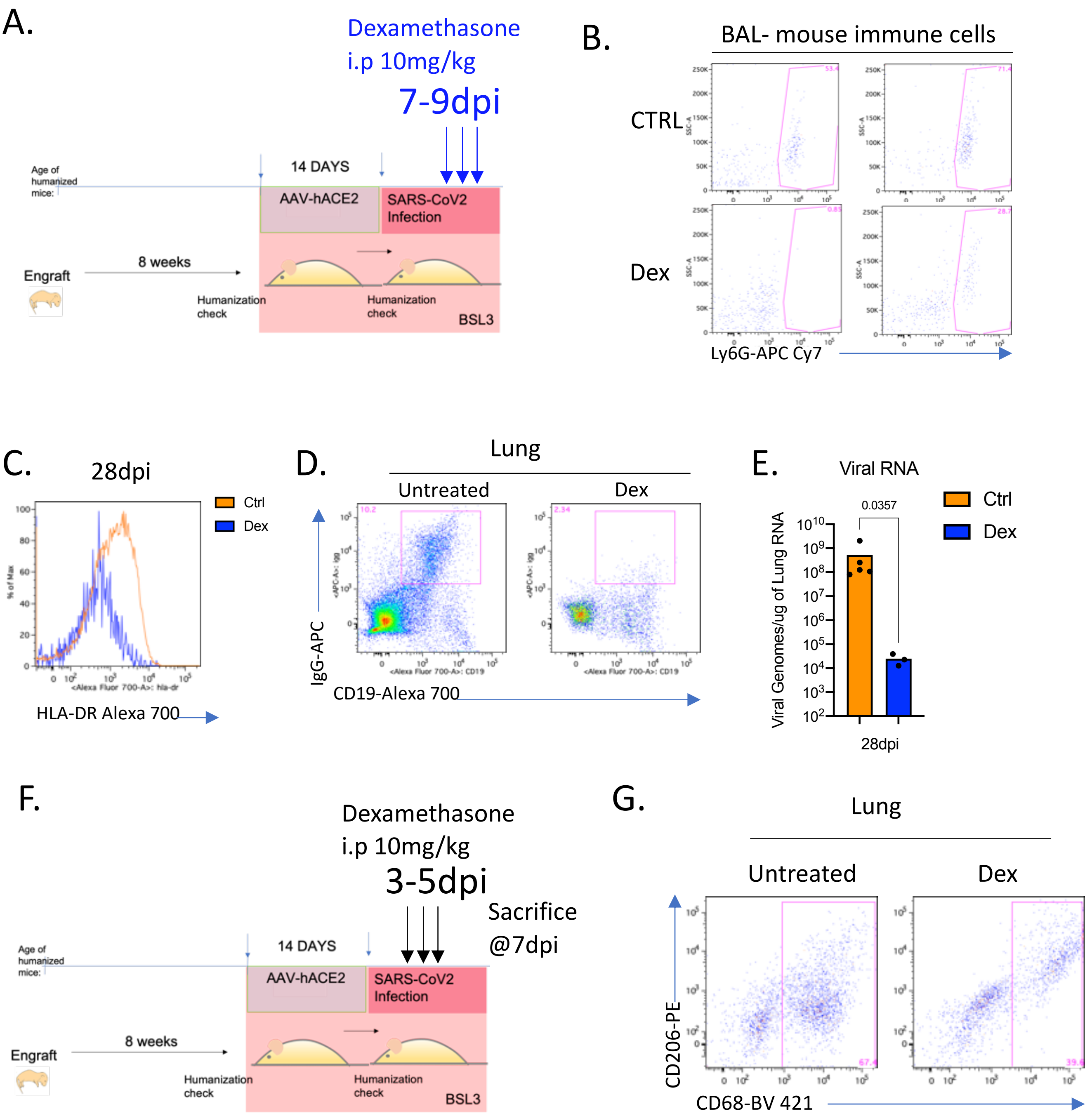

Figure S6:

A. Schematic of experimental design of SARS-Cov2 infected MISTRG6-hACE2 mice either treated with dexamethasone on days 7,8,9 dpi or left untreated.

B. Representative flow cytometry plots of Ly6G expressing SSChi cells within the mouse immune cell population (mouse CD45+) in the BAL of dexamethasone treated or control mice.

C. HLA-DR expression on lung T cells 28dpi in dexamethasone treated or control mice.

D. Representative flow cytometry plots of Surface IgG and CD19 expression on human immune cells gated on hCD45+ cells in lungs of untreated or dexamethasone treated mice at 28dpi. N=4

E. Viral RNA in the lung homogenates of dexamethasone treated or control untreated mice at 28dpi. N=3-5. Mann-Whitney, two-tailed test.

F. Schematic of experimental design of SARS-Cov2 infected MISTRG6-hACE2 mice either treated with dexamethasone on days 3,4,5 dpi or left untreated.

G. CD206 and CD68 expression in lung human immune cells in mice treated with dexamethasone or left untreated at 7dpi. CD206hi+ CD68+ cells are alveolar macrophages. N=4-6
